# Heme–Protein Interactions and Functional Relevant Heme Deformations: The Cytochrome c Case

**DOI:** 10.3390/molecules27248751

**Published:** 2022-12-09

**Authors:** Reinhard Schweitzer-Stenner

**Affiliations:** Department of Chemistry, Drexel University, Philadelphia, PA 19104, USA; rs344@drexel.edu; Tel.: +1-215-895-2268

**Keywords:** cytochrome c, structure and function, electronic structure, heme–protein interactions, symmetry-lowering deformations, redox potential, electron transfer

## Abstract

Heme proteins are known to perform a plethora of biologically important functions. This article reviews work that has been conducted on various class I cytochrome c proteins over a period of nearly 50 years. The article focuses on the relevance of symmetry-lowering heme–protein interactions that affect the function of the electron transfer protein cytochrome c. The article provides an overview of various, mostly spectroscopic studies that explored the electronic structure of the heme group in these proteins and how it is affected by symmetry-lowering deformations. In addition to discussing a large variety of spectroscopic studies, the article provides a theoretical framework that should enable a comprehensive understanding of the physical chemistry that underlies the function not only of cytochrome c but of all heme proteins.

## 1. Introduction

**Figure 1:**
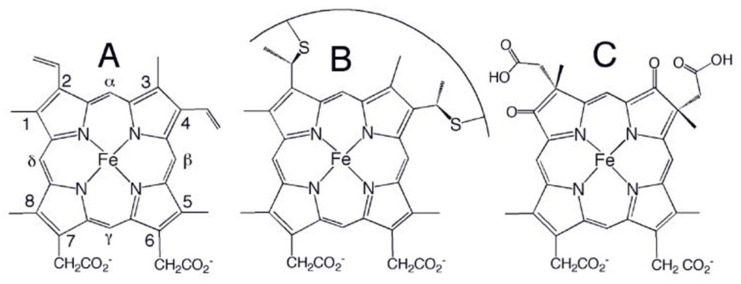
Structure of heme b ((protoporphyrin IX, (**A**)), heme c (**B**) and heme a (**C**). Reprinted with permission from [15], 2008, Royal Society of Chemistry.

The family of heme proteins plays a peculiar and very prominent role among the multitude of proteins identified and characterized thus far. They perform a diverse set of biochemical functions, such as ligand binding and transfer (myoglobin, hemoglobin [1,2], cytoglobin [3,4], neuroglobin [5]), electron transfer (coenzyme Q, cytochrome c, cytochrome c oxidase, reaction centers) [6,7,8,9] and enzymatic reactions of various types (horseradish peroxidase, guanylate cyclase, cytochrome P450 and lignin peroxidase as canonical representatives of the peroxidases, cyclases, mono-oxygenases and ligninases) [10,11]. These proteins differ in terms of their secondary structure composition and adopted tertiary (and sometimes quaternary) structures, but they have very similar actives sites in common, namely iron porphyrines, which differ mostly in regard to their peripheral substituents. Three representative examples are shown in Figure 1. Hemo- and myoglobin contain protoporphyrin IX (heme b, Figure 1) as an active site that exhibits an asymmetric arrangement of methyl, propionic acid and vinyl groups, each of which interacts non-covalently with the respective protein moiety [12]. In both cases, the imidazole group of the so-called proximal histidine coordinates with the heme iron. The sixth coordination site can be occupied by external ligands such as O_2_, CO, NO, OH, CN and H_2_O, depending on the iron’s oxidation state. Another prominent representative of the heme group family is heme c, which can be found in all proteins of the highly diverse cytochrome c family (Figure 1) [13,14]. In these proteins, the two vinyl groups of protoporphyrin IX are covalently linked to cysteins via thioether bridges. In most cases, the two cysteins belong to a highly conserved CxxCH motif, where x represents a variety of amino acid residues. The terminal histidine of the motif coordinates with the heme iron. In some cases, this motif contains more than two x-residues. In rare cases, only a single thioether bridge is formed. In class I cytochrome c proteins, the sixth ligand is provided by the sulfur atom of a methionine side chain, and the heme group is located near the N-terminal. The canonical mitochondrial cytochrome c is a member of this family. Classes of cytochrome c differ in terms of their secondary and tertiary structures and the number of incorporated heme groups. The third type of heme group, shown in Figure 1, is heme a, a functional group in, e.g., cytochrome c oxidase [8]. Here, one of the methyl groups of heme b is oxidized to a formyl group. One of the vinyl substituents is replaced by a hydroxyethylfarnesyl group. There are two heme a groups in cytochrome c oxidase, termed heme a and a3. Together with Cu_B_, the latter constitute a binuclear center which catalyzes the reduction of O_2_. The axial coordinates of heme a in cytochrome c oxidase are provided by two histidines, while the resting state of heme a3 is pentacoordinate, with an imidazole side chain as the axial ligand.

The biological function of proteins is frequently linked to structural changes. A prominent example is oxygen binding to hemoglobin, which triggers a change in the tertiary structure of the respective subunit [2,16]. This produces a mismatch between the two quaternary structures that reduces the Gibbs energy difference between them. Consecutive oxygen binding thus produces a switch between a low-affinity T and a high-affinity R-state [17,18,19,20,21,22]. By comparison, structural changes induced by the reduction in/oxidation of the heme iron in mitochondrial cytochrome c are moderate and mostly involve changes in the Fe-S(M) bond length, inter-residue hydrogen bonding and water orientations in the heme pocket [23,24,25,26,27,28,29]. However, the situation is different when the protein binds to anionic surfaces, such as the inner membrane of the mitochondria, in that this causes a conformational change which involves the replacement of the methionine ligand by the imidazole side chain of a histidine and, thus, a significant decrease in the reduction potential [13,14,30,31,32,33,34]. In this state, the protein acquires some moderate peroxidase activity [35]. On the contrary, structural changes involving interactions between cytochrome c and cytochrome c oxidase and the subsequent redox reactions in the latter are moderate and rather local [8,36,37].

**Figure 2:**
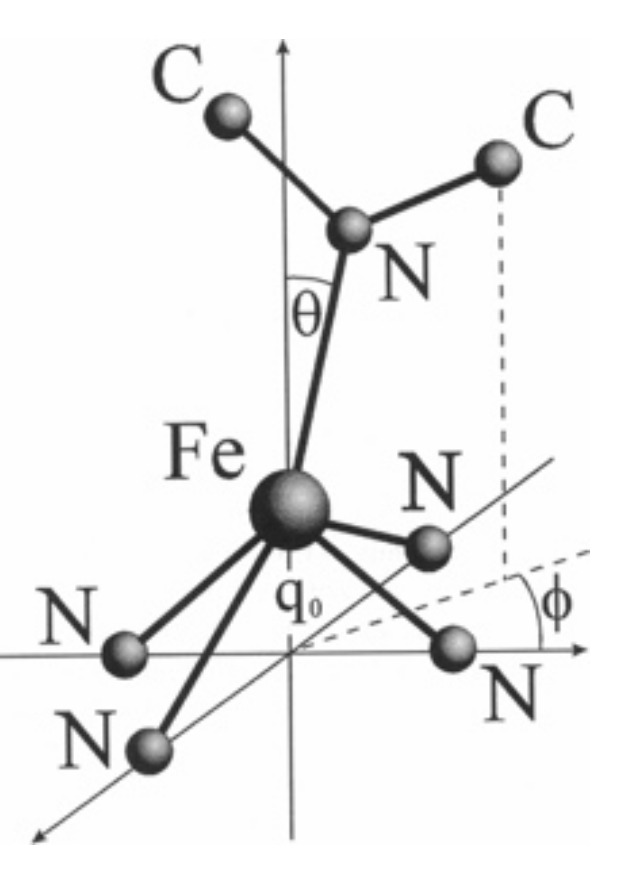
Schematic representation of the iron out-of-plane displacement of the heme group in deoxy-myoglobin and deoxyhemoglobin. Reprinted with permission from [52], 1993, Elsevier.

While function-related alterations in the structure of heme protein have been the focus of research activities since the necessary experimental tools became available (X-ray diffraction and, later, multidimensional NMR, resonance Raman and circular dichroism spectroscopy), changes in the porphyrin macrocycle have been given little attention for a longer period of time. In spite of the asymmetric arrangement of the peripheral substituents and a plethora of heme–protein contacts, the macrocycle of, e.g., hemes b and c were generally assumed to be planar and to exhibit a D_4h_ symmetry [38,39,40]. This seemed to be justifiable in view of its aromatic character and the assumed lack of electronic substituent–macrocycle interactions. The only deviation from the rule was the so-called iron out-of-plane displacement of pentacoordinate heme groups, with its metal in the reduced state (Fe^2+^) [41]. A binding of a second axial ligand was generally assumed to eliminate this displacement [2,42]. The schematic representation of this deformation in Figure 2 shows that it was assumed to involve solely the metal atom. However, X-ray structures of both oxidation states of mitochondrial cytochrome c proteins [26] and of the two subunit types of oxygenated hemoglobin clearly reveal that this view is by far too simplistic [43]. In the latter, the heme group maintains some of the domed structure that it assumes in the deoxygenated ferrous state of the protein. In both redox states of yeast iso-1-cytochrome c, the heme group exhibits a high degree of non-planarity (vide infra). Further evidence of a deviation from an ideal D_4h_ symmetry comes from nuclear magnetic (NMR) and electron paramagnetic resonance data (EPR), which, for cytochrome c, are clearly diagnostic of a highly asymmetric spin distribution and rhombic deformations of the metal’s crystal field [44,45,46,47]. Resonance Raman dispersion and visible circular dichroism spectroscopy clearly revealed the presence of electronic-deformation-inducing and vibronic perturbations [48,49,50,51].

In a biophysical context, the question arises as to whether these symmetry-lowering deformations of the heme group are functionally relevant. While several lines of evidence pointed in this direction for model porphyrins in solution [53], the functional significance, particularly that of out-of-plane deformations, have only been revealed more recently by the work of Bren, Walker and their respective associates [47,54,55,56]. This review puts their accomplishments into the broader context. For the sake of brevity and readability, I will focus on cytochrome c, which has been a laboratory fundamental for biophysical research since the pioneering work of Theorell and Åkesson in the 1940s of the last century. In this context, the review will focus mostly on class I cytochromes [57]. In what follows, Section 2 starts with a simple quantum mechanical model that describes the electronic structure of the heme groups in the presence of symmetry-lowering perturbations. In Section 3, the early EPR results are described and interpreted in terms of the symmetry of the metal’s ligand field. It will be shown that EPR is a suitable tool for probing the in-plane deformations of the heme group. In Section 4, the review turns to resonance Raman spectroscopy and shows how this technique can be utilized to determine the in-plane and out-of-plane deformations of the heme group. In Section 5, I focus on the normal mode structure decomposition method, by means of which Shelnutt and coworkers obtained out-of-plane deformations from the crystal structures of heme proteins (cf. [58] and references therein). Section 6 of the article discusses the work of Bren and colleagues, who explored the relationship between the heme deformations, NMR chemical shifts and redox potentials of cytochrome c derivatives (cf. [59] and references cited therein). The Summary and Outlook brings this review to a close.

## 2. Protein-Induced Symmetry-Lowering Perturbations

In what follows, I use an ideal porphyrin macrocycle in a D_4h_ symmetry as a reference system. The most general form of the time-independent Schrödinger equation can be written as:

(1) $$\hat{H}\left|\psi_{i}\left(r,q\right)\rangle =E_{i}\right|\psi_{i}\left(r,q\right)\rangle$$

where the Hamiltonian $\hat{H}$ contains the electronic and vibrational kinetic and potential energy. $\left|\psi_{i}\left(r,q\right)\right\rangle$ denotes the *ith* eigenfunctions, which depend on a set of electronic and nuclear coordinates, which I denote collectively as *r* and *q*. The latter can be identified with the the normal coordinates of the system.

For a zeroth-order approach, I invoke the crude Born–Oppenheimer approximation, which allows us to separate electronic and vibrational wavefunctions and express the former for the equilibrium geometry of the heme macrocycle. Hence, the electronic Schrödinger equation can be written as:

(2) $${\hat{H}}_{el}\left|\psi_{el,i}\left(r,q_{0}\right)\rangle =E_{el,i}\right|\psi_{el,i}\left(r,q_{0}\right)\rangle$$

where *q_0_* represents the set of normal coordinates at equilibrium, and the subscript *el* indicates that only electronic contributions are taken into account.

**Figure 3:**
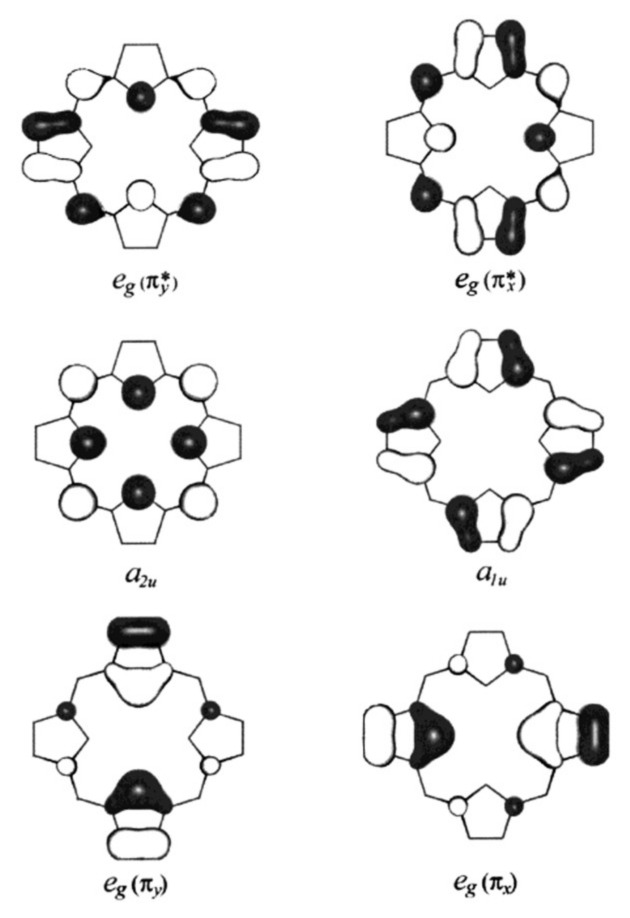
Frontier orbitals of a metal porphyrin in D_4h_ symmetry. The orbitals *3a_1u_*, *3a_2u_* and *3e_g_* are filled with electrons. The antibonding orbital *4e_g_* is the LUMO. While *3a_1u_* and *3a_2u_* always lie at higher energies than *3e_g_*, the hierarchy of the former depends on peripheral substituents and axial ligands. In an idealized reference system, they are assumed to be accidentally degenerate. Reprinted with permission from [60], 2003, American Chemical Society.

**Figure 4:**
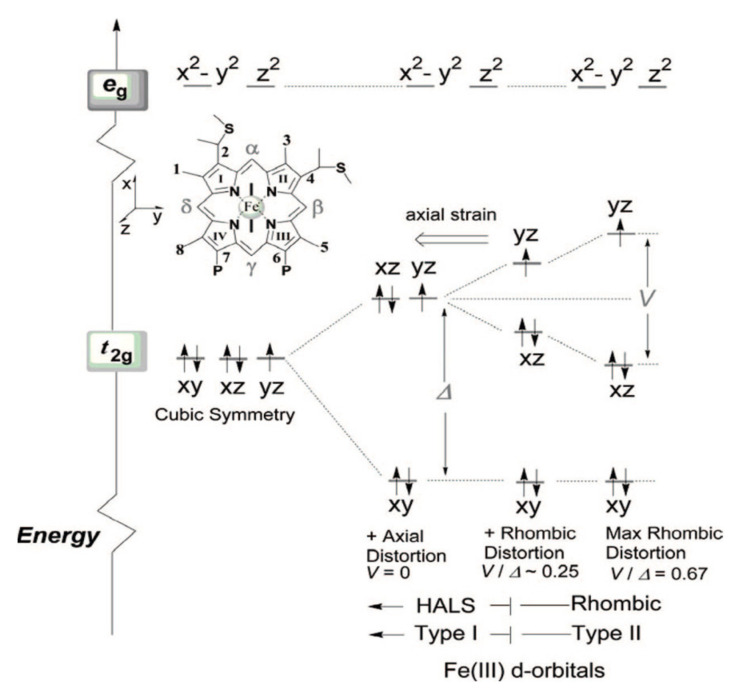
Energy diagram and orbital occupancy of a ferric iron in a strong ligand field that stabilizes a low spin configuration. For the sake or readability, the influence of the distortions that lower the symmetry from cubic to rhombic are only shown for the occupied orbitals. Note that the depicted hierarchy assumes that the *d_π_*-orbitals exhibit higher energies than the *d_xy_*-orbital. This is generally the case for the most prominent cytochrome c derivatives. HALS denotes a highly axial low-spin heme iron. Rhombic deformations of the heme core are discussed in detail in the text. Reprinted with permission from [47], 2008, American Chemical Society.

The classical four-orbital model of Gouterman describes the electronic structure of the porphyrin macrocycle in terms of two HOMOS of *A_1u_* and *A_2u_* symmetry (in what follows, I will use capital letters to denote symmetries (irreducible representations of point groups,) while lower-case letters indicate a specific molecular orbital) and a LUMO of *E_g_* symmetry (Figure 3). In a zeroth-order reference system, the two HOMOs are accidentally degenerate, but the configuration interaction lifts this degeneracy by a substantial amount. The model ignores the d-orbitals of the central iron atom, which are mostly discussed in the context of crystal or ligand field theories. As shown in Section 2, Section 3 and Section 4, such a separation is inconsistent with EPR and particularly with NMR data. In order to formulate a more holistic approach, I first remind the reader of the ligand field theory for Fe^2+^ and Fe^3+^ symmetry, which is illustrated in Figure 4. In an octahedral ligand field, the five d orbitals, which are degenerate in the absence of zero-field splitting, split into two groups of *E_g_* (higher energy) and *T_2g_* symmetry (lower energy). The splitting energy Δ_0_ depends on the strength of the ligand field. If the symmetry is lowered to D_4h_, further orbital splitting occurs, though to a different extent. The higher-lying *e_g_*-orbital splits into its $d_{z^{2}}$ (*A_1g_*) and $d_{x^{2}-y^{2}}$ (*B_1g_*) components, while the lower-lying *t_2g_*-orbital splits into $d_{\pi }$($d_{xz},d_{yz}$, *E_g_*) and $d_{xy}$ (*B_2_*_g_). The respective hierarchy depends on the ligand field. Figure 4 shows the energy level diagram generally obtained for ligand fields in class I cytochrome c derivatives. The $d_{z^{2}}$-orbital exhibits the highest energy. The twofold degenerate $d_{\pi }$-orbitals are higher in energy than $d_{xy}$.

As I will show below, iron d-orbitals can mix with porphyrin orbitals in the presence of the out-of-plane deformations of the latter. In the D_4h_ symmetry assumed thus far, the *e_g_*-orbitals of the macrocycle and the heme iron can mix. Regarding to the former, the occupied *3e_g_* and the unoccupied *4e*_g_-orbitals (LUMO) fall into this category [47]. Among the d-orbitals, the $d_{\pi }$ pair exhibits *E_g_* symmetry. NMR data provided evidence of some mixing of 3*e_g_* (Figure 3) and $d_{\pi }$, which produce spin density in the pyrrole rings of the macrocycle. On the contrary, interactions between $d_{\pi }$ and the *4e_g_* LUMO are negligible [47,54] Based on these findings, the classical four-orbital model of the porphyrin ring should be slightly extended to yield the following basis set of electron configurations:

(3) $$\left|\psi_{1}\rangle {=}^{2}\right|3a_{1u}^{2},3a_{2u}^{2},{\left(3e_{g}-d_{\pi }\right)}^{4},{\left(d_{\pi }+3e_{g}\right)}^{3},d_{xy}^{2}\rangle \;\left|\psi_{2}\rangle {=}^{2}\right|3a_{1u}^{2},3a_{2u}^{2},{\left(3e_{g}-d_{\pi }\right)}^{4},{\left(d_{\pi }+3e_{g}\right)}^{4},d_{xy}^{1}\rangle \;\left|\psi_{3}\rangle {=}^{2}\right|3a_{1u}^{2},3a_{2u}^{2},{\left(3e_{g}-d_{\pi }\right)}^{3},{\left(d_{\pi }+3e_{g}\right)}^{4},d_{xy}^{2}\rangle \;\left|\psi_{4}\rangle {=}^{2}\right|3a_{1u}^{1},3a_{2u}^{2},{\left(3e_{g}-d_{\pi }\right)}^{4},4e_{g}^{1},{\left(d_{\pi }+3e_{g}\right)}^{3},d_{xy}^{2}\rangle \;\left|\psi_{5}\rangle {=}^{2}\right|3a_{1u}^{2},3a_{2u}^{1},{\left(3e_{g}-d_{\pi }\right)}^{4},4e_{g}^{1},{\left(d_{\pi }+3e_{g}\right)}^{3},d_{xy}^{2}\rangle \;\left|\psi_{6}\rangle {=}^{2}\right|3a_{1u}^{1},3a_{2u}^{2},{\left(3e_{g}-d_{\pi }\right)}^{3},4e_{g}^{1},{\left(d_{\pi }+3e_{g}\right)}^{4},d_{xy}^{2}\rangle \;\left|\psi_{7}\rangle {=}^{2}\right|3a_{1u}^{2},3a_{2u}^{1},{\left(3e_{g}-d_{\pi }\right)}^{3},4e_{g}^{1},{\left(d_{\pi }+3e_{g}\right)}^{4},d_{xy}^{2}\rangle \;\left|\psi_{8}\rangle {=}^{2}\right|3a_{1u}^{1},3a_{2u}^{2},{\left(3e_{g}-d_{\pi }\right)}^{4},4e_{g}^{1},{\left(d_{\pi }+3e_{g}\right)}^{4},d_{xy}^{1}\rangle \;\left|\psi_{9}\rangle {=}^{2}\right|3a_{1u}^{2},3a_{2u}^{1},{\left(3e_{g}-d_{\pi }\right)}^{4},3e_{g}^{1},{\left(d_{\pi }+3e_{g}\right)}^{4},d_{xy}^{1}\rangle$$

Here, the notations *3e_g_* − $d_{\pi }$ and *3e_g_* + $d_{\pi }$ simply indicate an out-of-phase and in-phase combination of the two orbitals. The superscript in front of the ket indicates that all these states are doublets. The states have been numbered so that they line up with the expected increase in their eigenenergies. Quartet states, which involve the transition of an electron from $d_{\pi }$ into $d_{z^{2}}$ or $d_{x^{2}-y^{2}}$ can be expected to have an energy too high to be thermally populated at room temperature.

It should be noted that this model is still somewhat simplistic for two reasons. First, it ignores the mixture of the metal d- with π-orbitals of the axial ligands. In the case of cytochrome c, we thus neglect the mixing with the imidazole π-orbitals and the lone pair of methionine sulfur (vide infra), which can have a measurable influence on the eigenenergies of $\left|\psi_{2}\right\rangle$ and $\left|\psi_{3}\right\rangle$ [56,59,61]. Secondly, it ignores the influence of spin orbit coupling, which mixes the $d_{\pi }$- and $d_{xy}$-orbitals [44]. Within the basis set defined by $\left|\psi_{1}\right\rangle$….$\left|\psi_{9}\right\rangle$, the electronic Hamiltonian can be written as follows:

(4) $$H=\left(\begin{array}{ccccccccc} E_{1} & 0 & 0 & 0 & 0 & 0 & 0 & 0 & 0\\ 0 & E_{2} & 0 & 0 & 0 & 0 & 0 & 0 & 0\\ 0 & 0 & E_{3} & 0 & 0 & 0 & 0 & 0 & 0\\ 0 & 0 & 0 & E_{4} & \delta_{45}/2 & 0 & 0 & 0 & 0\\ 0 & 0 & 0 & \delta_{45}/2 & E_{5} & 0 & 0 & 0 & 0\\ 0 & 0 & 0 & 0 & 0 & E_{6} & \delta_{67}/2 & 0 & 0\\ 0 & 0 & 0 & 0 & 0 & \delta_{67}/2 & E_{7} & 0 & 0\\ 0 & 0 & 0 & 0 & 0 & 0 & 0 & E_{8} & \delta_{89}/2\\ 0 & 0 & 0 & 0 & 0 & 0 & 0 & \delta_{89}/2 & E_{9} \end{array}\right)$$

where the diagonal elements are the eigenenergies of the wave functions of Equation (3) in the absence of the configurational interactions (CI) δ. The different subscripts of δ indicate that CI might be different for configurations with different electron occupations of 3e_g_ − $d_{\pi }$, 3e_g_ + $d_{\pi }$ or $d_{xy}$. Owing to the block character of the Hamiltonian matrix, diagonalization is straightforward and leads to the following new basis set. Here, one must be mindful of the fact that the matrix elements of configuration interactions are two-electron integrals. Hence, they couple (*a_2u_*,*e_gx_*) with (*a_1u_*,*e_gy_*), and vice versa. With this in mind, and by neglecting the very high lying states $\left|\psi_{7}\right\rangle$–$\left|\psi_{9}\right\rangle$, one obtains the following new basis set:

(5) $$\left|g_{0x}\rangle =\right|\psi_{1x}\rangle {=}^{2}|{\left(d_{xz}+3e_{gx}\right)}^{3}\rangle \;\left|g_{0y}\rangle =\right|\psi_{1y}\rangle {=}^{2}|{\left(d_{yz}+3e_{gy}\right)}^{3}\rangle \;\left|g_{1}\rangle =\right|\psi_{2}\rangle {=}^{2}|{\left(d_{\pi }+3e_{g}\right)}^{4},d_{xy}^{1}\rangle \;\left|g_{2x}\rangle =\right|\psi_{3x}\rangle {=}^{2}|{\left(3e_{gx}-d_{xz}\right)}^{3},{\left(d_{xz}+e_{gx}\right)}^{4}\rangle \;\left|g_{2y}\rangle =\right|\psi_{3y}\rangle {=}^{2}|{\left(3e_{gy}-d_{yz}\right)}^{3},{\left(d_{yz}+e_{gy}\right)}^{4}\rangle \;\left|Q_{x}^{0}\rangle =sin\nu \cdot^{2}\right|3a_{2u}^{1},4e_{gx}^{1},{\left(d_{\pi }+3e_{g}\right)}^{3}\rangle +cos\nu \cdot^{2}|3a_{1u}^{1},4e_{gy}^{1},{\left(d_{\pi }+3e_{g}\right)}^{3}\rangle \;\left|Q_{y}^{0}\rangle =cos\nu \cdot^{2}\right|3a_{2u}^{1},4e_{gy}^{1},{\left(d_{\pi }+3e_{g}\right)}^{3}\rangle -sin\nu \cdot^{2}|3a_{1u}^{1},4e_{gx}^{1},{\left(d_{\pi }+3e_{g}\right)}^{3}\rangle \;\left|B_{x}^{0}\rangle =cos\nu \cdot^{2}\right|3a_{2u}^{1},4e_{gx}^{1},{\left(d_{\pi }+3e_{g}\right)}^{3}\rangle -sin\nu \cdot^{2}|3a_{1u}^{1},4e_{gy}^{1},{\left(d_{\pi }+3e_{g}x\right)}^{3}\rangle \;\left|B_{y}^{0}\rangle =sin\nu \cdot^{2}\right|3a_{2u}^{1},4e_{gy}^{1},{\left(d_{\pi }+3e_{g}\right)}^{3}\rangle +cos\nu \cdot^{2}|3a_{1u}^{1},4e_{gx}^{1},{\left(d_{\pi }+3e_{g}\right)}^{3}\rangle$$

For the sake of brevity, I use only the not fully occupied and excited orbitals in the notation for the electron configurations of the considered states. The mixing parameter ν relates to the coupling energy matrix element as follows:

(6) $$\nu =\frac{1}{2}arctan\left(\frac{2\cdot \delta_{45}}{E_{5}-E_{4}}\right)$$

Apparently, if *E_4_* = *E_5_* (i.e., *3a_1u_* and *3a_2u_* are accidentally degenerate), *ν*_ij_ = π/4 [20]. In the literature, this state is often chosen as a reference state [62]. Any lifting of the degeneracy is then ascribed to an *A_1g_*-type perturbation [16].

**Figure 5:**
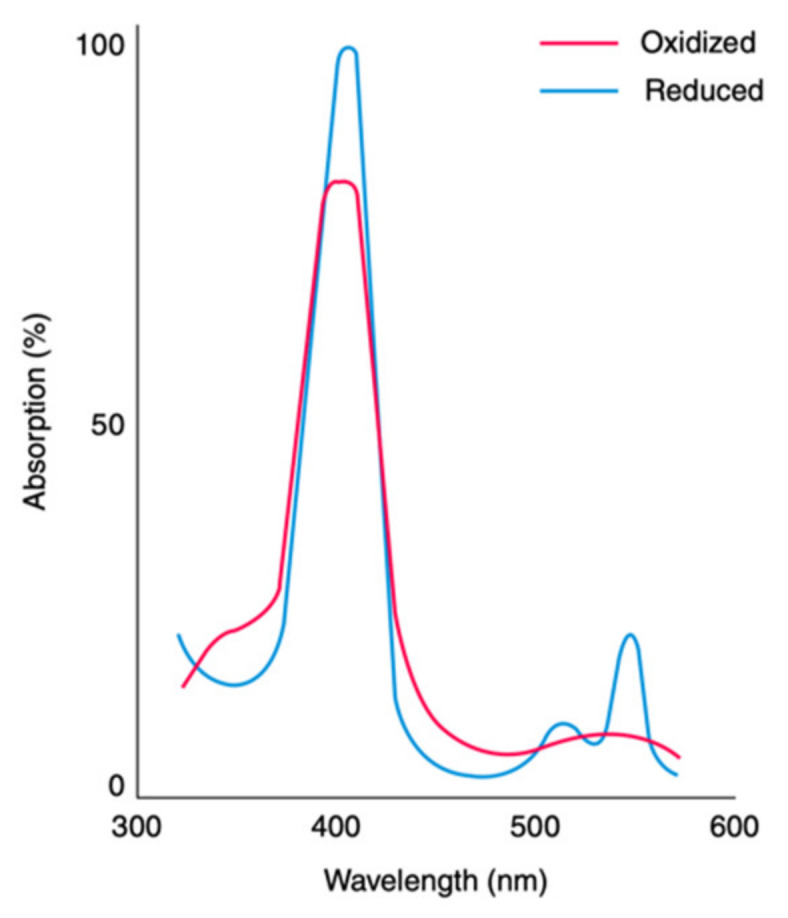
UV–Vis absorption spectra of oxidized and reduced cytochrome c. Reprinted with permission from [66], 2012, Elsevier.

The absorption spectra of cytochrome c and other heme proteins (Figure 5) contain a very intense band in the region between 410 and 430 nm and a weak band in the 550 nm region. The former is called Soret or B-band, and the latter is a superposition of the so-called α- and β-band, which are termed Q_0_ and Q_v_ in the spectroscopic literature. These two bands are clearly resolved in the spectrum of ferrocytochrome c but merge into a single, broad optical band in the spectrum of the oxidized protein. However, as shown by Dragomir et al., they are still distinguishable in the respective circular dichroism spectrum [49]. The B-band is generally assigned to a $\left|g_{0x,y}\rangle \to \right|B_{x,y}\rangle$ transition, whereas the Q_0_-band is understood to result from $\left|g_{0x,y}\rangle \to \right|Q_{x,y}\rangle$. Both transitions are electronically dipole-allowed (*vide infra*). The much weaker intensity of the Q_0_-band results from the configurational interaction between the states $\left|\psi_{6}\right.\;\left(\left|\psi_{7}\right.\right)$ and $\left|\psi_{8}\right.\;\left(\left|\psi_{9}\right.\right)$ in Equation (3) [62]. The Q_v_-band results from vibronic coupling between $\left|B_{x,y}\right\rangle$ and $\left|Q_{x,y}\right\rangle$, which involves the excitation of vibrational states in $\left|Q_{x,y}\right\rangle$ [63]. The B-band has a vibronic side band B_v_, which overlaps with the B_0_-band. It mostly originates from Franck–Condon-type transitions into the first vibrational state of totally symmetric porphyrin modes [64,65].

Traditionally, within the framework of the four-orbital model, the electronic transitions $\left|\psi_{1}\rangle \to \right|\psi_{4}\rangle$ and $\left|\psi_{1}\rangle \to \right|\psi_{5}\rangle$ are described as *A_1_*_g_(*a_1u_^2^*, *a_2u_^2^*)→*E_u_*(*a_1u_^1^*,*e_g_^1^*,*a_2u_^2^*) and *A_1g_*(*a_1u_^2^*, *a_2u_^2^*)→*E_u_*(*a_1u_^2^*,*e_g_^1^*,*a_2u_^1^*). Both singlet transitions are electronically allowed. If one considers the above extended electronic configuration instead, the ground state is a doublet and transforms in the same manner as *E_g_* in D_4h_. The symmetry of the excited-state configuration could be written as *E_g_* × *E_g_* = *A_1u_* + *A_2u_* + *B_1u_* + *B_2u_*. Since the ground state transforms in the same manner as *E_g_*, the electronic transition dipole moment has *E_u_* symmetry, as in the case of the four-orbital model.

Now, I consider a more realistic scenario, where an electronic perturbation term ${\hat{V}}_{el}^{\Gamma }$ is added to the Schrödinger equation 2, which is now written as [67,68]:

(7) $$\left({\hat{H}}_{el,0}+{\hat{V}}_{el}^{\Gamma }\right)\left|\psi_{i}^{’’}\left(r,q\right)\rangle =E_{i}^{’’}\right|\psi_{i}^{’’}\left(r,q\right)\rangle$$

where *Γ* denotes the symmetry of an irreducible representation in D_4h_. Hence, it is assumed that the perturbing potential can be deconstructed into the symmetries of this point group. The matrix representation of the Hamiltonian is the following:

(8) $$H_{el}=\left(\begin{array}{ccccccccc} E_{g0x}+V^{B_{1g}} & V^{B_{2g}} & V^{B_{1g}} & 0 & 0 & 0 & 0 & 0 & 0\\ V^{B_{2g}} & E_{g0y}-V^{B_{1g}} & 0 & -V^{B_{1g}} & 0 & 0 & 0 & 0 & 0\\ V^{B_{1g}} & 0 & E_{g1x}+V^{B_{1g}} & V^{B_{2g}} & 0 & 0 & 0 & 0 & 0\\ 0 & -V^{B_{1g}} & V^{B_{2g}} & E_{g1y}-V^{B_{1g}} & 0 & 0 & 0 & 0 & 0\\ 0 & 0 & 0 & 0 & E_{g2} & 0 & 0 & 0 & 0\\ 0 & 0 & 0 & 0 & 0 & E_{Q_{0x}}+V^{B_{1g}} & V^{B_{2g}} & V^{B_{1g}} & V^{B_{2g}}+V^{A_{2g}}\\ 0 & 0 & 0 & 0 & 0 & V^{B_{2g}} & E_{Q_{0y}}-V^{B_{1g}} & V^{B_{2g}}-V^{A_{2g}} & -V^{B_{1g}}\\ 0 & 0 & 0 & 0 & 0 & V^{B_{1g}} & V^{B_{2g}}-V^{A_{2g}} & E_{B_{0x}}+V^{B_{1g}} & V^{B_{2g}}\\ 0 & 0 & 0 & 0 & 0 & V^{B_{2g}}+V^{A_{2g}} & -V^{B_{1g}} & V^{B_{2g}} & E_{B_{0y}}-V^{B_{1g}} \end{array}\right)$$

Apparently, the matrix of the Hamiltonian contains only perturbations of *gerade* symmetry, which lead to the in-plane deformation of the heme macrocycle. Owing to the mixing of the *3e_g_* porphyrin orbitals with the threefold occupied $d_{\pi }$ orbital, the electronic ground state configuration exhibits *E_g_* symmetry, which is typical of many low-spin ferric systems [47,53]. This allows the matrix elements of $V^{\Gamma },\Gamma =B_{1g},B_{2g}$ to contain non-vanishing matrix elements in the ground state. This will be of relevance for the discussion of the EPR results on the oxidized cytochrome.

The matrix elements of $V^{\Gamma }$ of the excited states $\left|Q_{i}^{0}\rangle ,\right|B_{i}^{0}\rangle ,i=x,y$ deserve some further comments. The four-orbital model dictates that the electronic perturbations of *A_1g_*, *B_1g_*, *B_2g_* and *A_2g_* symmetry can mix the Q- and B-states, since *E_u_* × *E_u_* = *A_1g_* + *B_1g_* + *B_2g_* + *A_2g_.* While *B_1g_* and *B_2g_* deformations can produce intrastate (between the two Q- and the two B-states, respectively) and interstate coupling (between the Q- and B-states), *A_2g_* perturbations solely mix the Q- and B-states [16,69]. This scheme seems to become invalidated for this basis set, described in Equation (5), since the excited-state configuration no longer transforms in the manner of *E_u_.* However, if one considers only the perturbations that affect the electronic structure of the macrocycle, the matrix elements of $V^{\Gamma }$ can be simplified as follows. In a first step, the expressions for the Q and B can be rewritten as follows:

(9) $$\left|Q_{x}\rangle =\left(sin\nu \cdot \left|3a_{2u}^{1},4e_{gx}^{1}\rangle +cos\nu \cdot \right|3a_{1u}^{1},4e_{gy}^{1}\rangle \right)\cdot^{2}\right|{\left(d_{\pi }+3e_{g}\right)}^{3}\rangle \;\left|Q_{y}\rangle =\left(cos\nu \cdot \left|3a_{2u}^{1},4e_{gy}^{1}\rangle -sin\nu \cdot \right|3a_{1u}^{1},4e_{gx}^{1}\rangle \right)\cdot^{2}\right|{\left(d_{\pi }+3e_{g}\right)}^{3}\rangle \;\left|B_{x}\rangle =\left(cos\nu \cdot \left|3a_{2u}^{1},4e_{gx}^{1}\rangle -sin\nu \cdot \right|3a_{1u}^{1},4e_{gy}^{1}\rangle \right)\cdot^{2}\right|{\left(d_{\pi }+3e_{g}\right)}^{3}\rangle \;\left|B_{y}\rangle =\left(sin\nu \cdot \left|3a_{2u}^{1},4e_{gy}^{1},\rangle +cos\nu \cdot \right|3a_{1u}^{1},4e_{gx}^{1}\rangle \right)\cdot^{2}\right|{\left(d_{\pi }+3e_{g}\right)}^{3}\rangle$$

Now, the matrix elements of $V^{\Gamma }$ have the forms:

(10) $$\left\langle Q_{i}\left|V^{\Gamma }\right|Q_{j}\rangle =-\sin2\nu \langle Q_{i}^{0}|V^{\Gamma }|B_{j}^{0}\right\rangle \;\left\langle B_{i}\left|V^{\Gamma }\right|B_{j}\rangle =\sin2\nu \langle Q_{i}^{0}|V^{\Gamma }|B_{j}^{0}\right\rangle \;\left\langle Q_{i}\left|V^{\Gamma }\right|B_{j}\rangle =\cos2\nu \langle Q_{i}^{0}|V^{\Gamma }|B_{j}^{0}\right\rangle$$

where $\left|Q_{x}^{0}\rangle ,\right|Q_{y}^{0}\rangle ,\left|B_{x}^{0}\rangle ,\right|B_{y}^{0}\rangle$ represent the four canonical electron configurations of the four-orbital model in the absence of *A_1g_*-type deformations. This state is generally characterized as 50:50 mixing. The subscripts *i* and *j* represent *x* and *y.* The mixed heme-iron orbital function does not appear in Equation (10) because of their orthonormality.

If the electronic perturbation affects the porphyrin-iron orbital as well, one can proceed as follows. The eigenfunctions of the Hamiltonian in Equation (8) can be obtained by diagonalizing the two blocks formed by the sub-space states $\left|g_{0x}\rangle ,\right|g_{0y}\rangle ,\left|g_{1x}\rangle ,\right|g_{1y}\rangle$ and $\left|Q_{x}\rangle ,\right|Q_{y}\rangle ,\left|B_{x}\rangle ,\right|B_{y}\rangle$, respectively. Alternatively, if the interstate coupling is weak, one can diagonalize two-dimensional blocks formed by the degenerate states and treat the remainder of the Hamiltonian matrix with Rayleigh–Schrödinger perturbation theory. For both procedures, one would assume that the considered perturbations affect only the four porphyrin orbitals of the excited-state configuration (cf. Equations (9) and (10)). Next, one would consider the excited-state subset of the obtained eigenfunctions, which are now augmented by the above excluded porphyrin-iron orbital.

(11) $$|Q_{i}^{’}\rangle =\underset{j}{\sum }\left(\alpha_{QQ,ij}\left|Q_{j}\rangle \right|d_{jz}+3e_{gj}\rangle +\alpha_{QB,ij}\left|B_{j}\rangle \right|d_{jz}+3e_{gj}\rangle \right)\;i\;|B_{i}^{’}\rangle =\underset{j}{\sum }\left(\alpha_{BB,ij}\left|B_{j}\rangle \right|d_{jz}+3e_{gj}\rangle +\alpha_{BQ,ij}\left|Q_{j}\rangle \right|d_{jz}+3e_{gj}\rangle \right)$$

where *α_QQ,ij_* (*i,j*=x,y) are the eigenvectors of the new eigenstates. Equation (11) reflects the fact that in D_4h_ symmetry, the *3e_g_* + $d_{\pi }$ orbital and the four excited electronic porphyrin states share the same coordinate system with x and y along the Fe-N bonds and z perpendicular to the heme plane. Some of the symmetry-lowering perturbations considered in Equation (9) (i.e., those of *B_2g_* and *A_2g_* symmetry) cause a rotation of the coordinate system. I omit the multiplicity in Equation (11), since the exact electron configuration depends on the hierarchy of the two *d_π_* + *3e_g_* orbitals.

Let us now consider contributions to *V^Γ^*, which affect only the $d_{\pi }+3e_{g}$ orbital. In this case, it is sufficient to consider the following two-dimensional basis set:

(12) $$\left|\psi_{1x}^{FeP}\rangle =\right|d_{xz}+3e_{gx}\rangle \;\left|\psi_{1y}^{FeP}\rangle =\right|d_{yz}+3e_{gy}\rangle$$

Note that Equation (12) is formulated for the coordinate system of the unperturbed porphyrin. The matrix of the Hamiltonian accounting for the perturbing potential can thus be written as:

(13) $$V_{FeP}=\left(\begin{array}{cc} V_{FeP}^{B_{1g}} & V_{FeP}^{B_{2g}}\\ V_{FeP}^{B_{2g}} & -V_{FeP}^{B_{1g}} \end{array}\right)$$

I will discuss the relevance of the above formalism when I present the EPR, NMR circular dichroism and resonance Raman data below. Here, I only indicate that *B_1g_*-type perturbations split the Q- as well as the B-band transition. *A_2g_* and *B_2g_* perturbations can do the same if they occur together. Moreover, *B_1g_* perturbations lift the degeneracy of $\left|\psi_{x}^{FeP}\right\rangle$ and $\left|\psi_{y}^{FeP}\right\rangle$.

Thus far, I have solely discussed how an asymmetric potential produced by ligands and the protein environment can affect the electronic structure of the entire heme group. As Zgierski and Pawlikowski pointed out nearly 40 years ago, electronic perturbations must be distinguished from vibronic perturbations [68], which will be discussed in more detail below. They must be further distinguished from symmetry-lowering deformations of the heme group, which will be briefly discussed below.

The current discussion of heme (porphyrin) deformations relies on the normal-mode- based concept developed by Shelnutt, Jentzen and coworkers nearly 25 years ago [58,70,71,72,73]. It is called normal coordinate structural decomposition (NCD). These authors mostly focused on out-of-plane deformations, but, clearly, it is equally suitable for in-plane deformations. A similar approach was earlier introduced by Schweitzer-Stenner, Dreybrodt and their associated colleagues for the interpretation of the depolarization ratio dispersion of resonance Raman bands (vide infra) [74]. Until the work of Shelnutt and Jentzen appeared, these authors focused entirely on in-plane deformations.

The NCD approach is based on the assumption that any deformation of the heme group that is not excessively large can be understood as a linear combination of deformations along normal coordinates:

(14) $$\delta q_{total}=\sum_{\Gamma }\sum_{i}d_{i}^{\Gamma }\delta q_{i}^{\Gamma }$$

where *i* denotes the considered normal coordinate, and *Γ* is the irreducible representation in an ideal D_4h_ symmetry. A 24-atom porphyrin macrocycle has 21 out-of-plane and 45 in-plane modes. The symmetries of the former are *A_1u_*, *B_1u_*, *B_2u_*, *A_2u_* and *E_g_*, whereas in-plane modes can be classified in terms of *A_1g_*, *B_1g_*, *B_2g_*, *A_2g_* and *E_u_.* At first glance, the large number of normal coordinates might discourage a decomposition based on Equation (14) owing to the large number of options that such a large basis set can be expected to offer. However, a closer look at the origin of these distortions reveals that options are rather restricted. A distortion along the normal coordinates of a given symmetry in the electronic ground state can be calculated as follows [75]:

(15) $$\Delta_{g}^{\Gamma }=\sum_{k\left(\Gamma \right)}\frac{\left\langle g|\frac{\partial H_{el,0}\left(r,q\right)}{\partial q_{k}^{\Gamma }}|g\right\rangle }{{\left(\Omega_{k}^{g}\right)}^{2}}$$

The denominator contains the expectation value of the vibronic coupling operator for the *kth* mode of symmetry *Γ* in D_4h_ and wavenumber $\Omega_{k}^{g}$ in the electronic ground state |*g*>. Equation (15) reveals that the individual displacement along a given normal mode scales with the inverse of the square of its wavenumber in the ground state. Hence, only low-wavenumber modes contribute significantly to the deformations of the macrocycle of a given symmetry. Regarding out-of-plane modes, considering the lowest wavenumber vibration of a representation often suffices to describe the heme (porphyrin) deformation [76].

In the framework of the canonical four-orbital model, the ground state transforms in the manner of *A_1g_*. However, the mixture of *d_xz_*/*d_yz_* orbitals with *3e_g_* produces a ground state of *E_g_* symmetry. In D_4h_ symmetry, the *B_1g_* and *B_2g_* modes can induce Jahn–Teller (JT)-type coupling that produce Jahn–Teller distortions of the same symmetry. Apparently, *A_1g_*-modes can produce distortions as well, but obviously, they would not alter the symmetry of the macrocycle.

Apparently, JT coupling in the electronic ground state should predominantly involve heme vibrations with an amplitude at the pyrrole nitrogens (in-plane modes do not involve the metal). As shown below, the low-wavenumber modes of the *A_1g_*-, *B_1g_*- and *B_2g_*-modes meet this requirement.

The above considerations apply to the first part of the electronic Hamiltonian (i.e., ${\hat{H}}_{el,0}$) described in Equation (8), which transforms as *A_1g_* in D_4h_. Hence, the symmetry of the vibrations determines the symmetry of the vibronic coupling operator. However, if we consider the second term in Equation (8), the corresponding matrix elements are [16,63]:

(16) $$V_{el,gg}^{\Gamma x\Gamma^{\prime }}=\langle g\left|\partial V_{el}^{\Gamma }/\partial q_{k^{\Gamma^{\prime }}}\right|g\rangle$$

Group theoretically, all the combinations of *Γ* and *Γ’* for which the product representation is *gerade* are allowed. This includes the combination *Γ*=*Γ’* = *E_u_*. Following Ziergski and Pawlikowski, I term the derivative in Equation (16) as a vibronic perturbation [68]. In the presence of out-of-plane electronic perturbations (*Γ* = *A_1u_*, *B_1u_*, *B_2u_*, *A_2u_* and *E_g_*), all the out-of-plane modes can, in principle, contribute to the matrix element in Equation (16). The respective deformations scale, again, with the inverse of the square of their wavenumber in the electronic ground state, so that only low-wavenumber modes can be expected to contribute significantly to the total deformation of the heme group.

**Figure 6:**
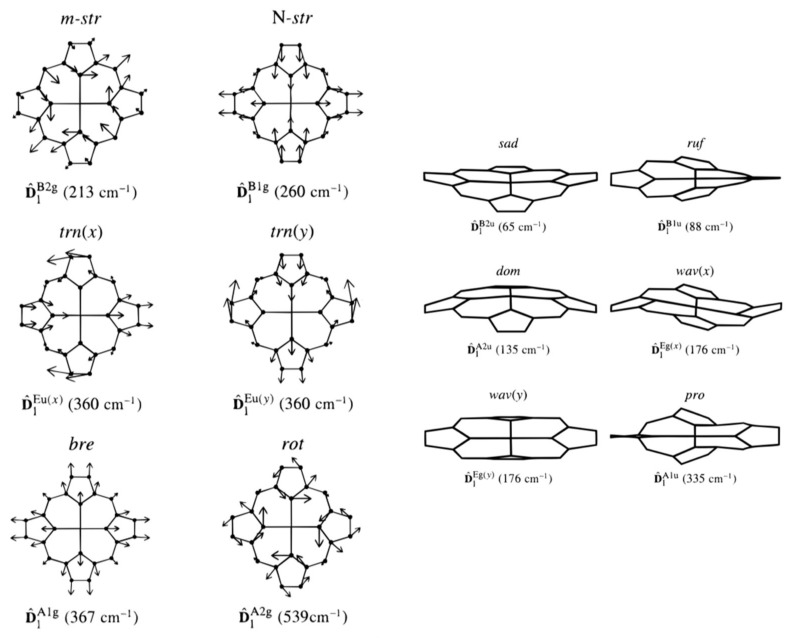
Representation of out-of-plane (right) and in-plane deformations (left) associated with the lowest-wavenumber normal mode of different symmetries in a D_4h_ symmetry. Reprinted with permission [70], 1997, American Chemical Society.

Figure 6 exhibits the deformations associated with the lowest-wavenumber modes of *A_1g_*, *B_1g_*, *B_2g_*, *E_u_*, *A_1u_*, *A_2u_*, *B_1u_*, *B_2u_* and *E_g_* symmetry. I neglect the respective *A_2g_*-mode because of its relatively high wavenumber [40,77]. I follow Jentzen et al. by using the following terminology for these deformations: breathing (*A_1g_*), stretching (*B_2g_*) translation (*E_u_*), propellering (*A_1u_*), doming (*A_2u_*), saddling (*B_2u_*), ruffling (*B_1u_*) and waving (*E_g_*) [70]. For *B_1g_*, I prefer the term rhombic so as to connect it to the interpretation of the EPR results (vide infra) [44].

## 3. EPR on Ferricytochrome c

Over the last 60 years, a large number of papers have reported results of the EPR measurements of heme proteins, in general, and cytochrome c derivatives, in particular. This is not the place for a comprehensive review of this vast amount of literature. Here, I focus on some representative papers that elucidate the deformations of the metal’s ligand field in the cytochrome c derivatives.

I start with the classical study of Salmeen and Palmer on beef heart ferricytochome c [44]. Since both the reduced and oxidized state of the metal are low-spin, only the latter has an unpaired electron and can be explored by EPR. The authors measured the EPR spectrum at 20 K with the X-band frequency. Their measurements revealed three different values for the elements of the diagonal g-tensor, namely 1.24, 2.24. and 3.06. The indicated anisotropy strongly suggests that the influence of axial and rhombic deformations do not only split the low-lying ^2^T_2g_ state (t_2g_^5^) of the octahedral symmetry into three Kramers doublets. In a first step, the authors proposed an energy scheme in which axial distortions lower the symmetry to D_4h_ (or C_4h_, if the two ligands are different) and split the *t_2g_*-orbitals (Figure 4). Salmeen and Palmer deduced from their data a higher-lying *d_xy_* (*B_2g_*)-orbital and a lower-lying twofold degenerate set of *d_xz_*- and *d_yz_*-orbitals, which would produce a ^2^*B_2g_* ground state. However, at the end of their paper, they revised their energy hierarchy in light of the results of the semi-empirical calculations of Zerner et al. [78] that put the two *d_π_*-orbitals above *d_xy_* to produce an *^2^E_g_* ground state (vide supra), as shown in Figure 4. In addition to axial and rhombic distortions, the authors considered the spin-orbit coupling of the three occupied orbitals, which, to some extent, allowed them to theoretically reproduce the experimental g-tensor values.

In a later study, Brautigan et al. showed that g-tensor values are indicative of rhombic deformations in horse heart and yeast *iso-1* oxidized cytochrome c [46]. The main purpose of their study was the evaluation of the crystal field as a function of pH. In the oxidized state, cytochrome c can adopt five to six different conformations if the pH is changed between 1 and 12 [57,79,80,81,82,83,84]. Since these changes are associated with changes in the sixth axial ligand (M80), the g-values of these conformations are substantially different.

**Figure 7:**
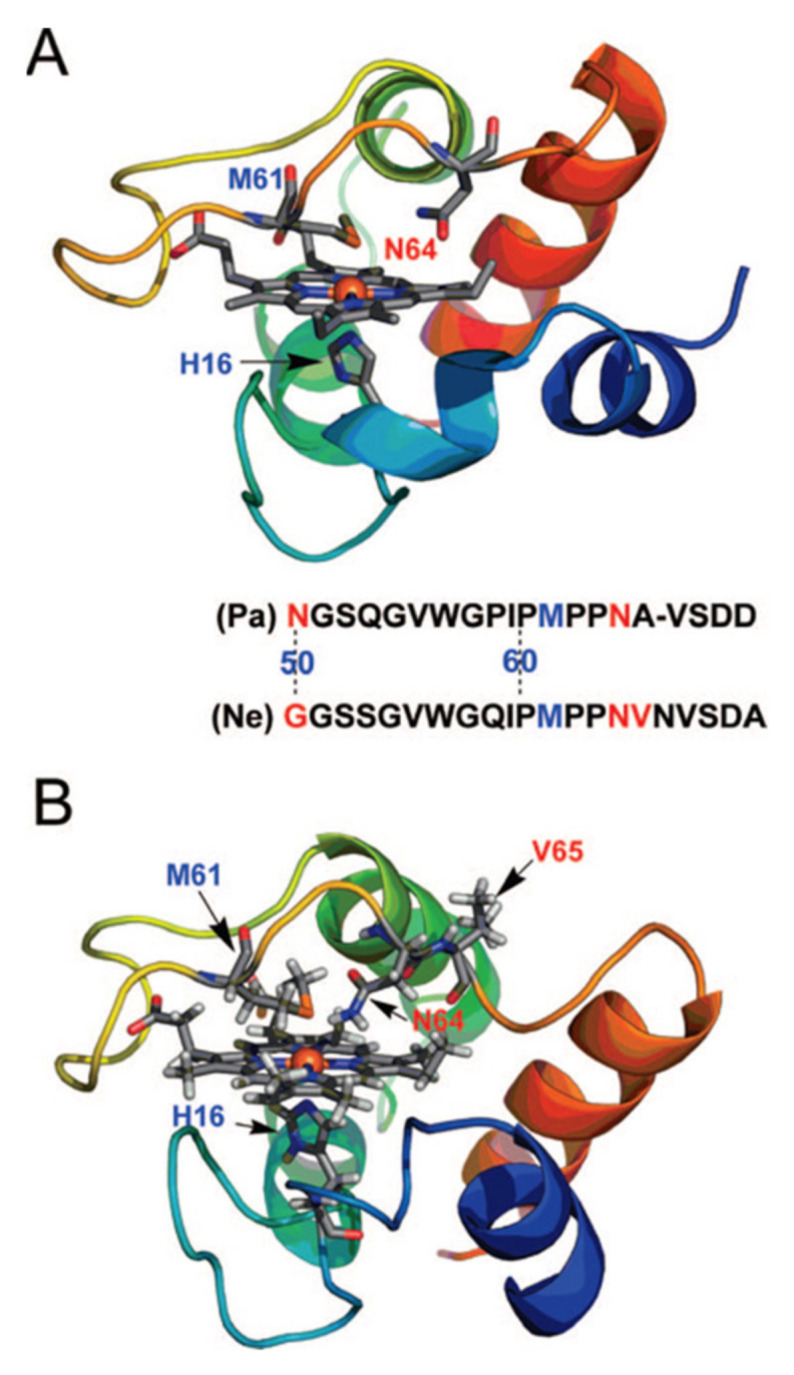
Structure of (**A**) Pa-*cyt_551_* (PDB code 351c) and (**B**) Ne *cyt_552_* (PDB code 1A56). The amino acid residues colored in red in the depicted sequences indicate point mutations. Reprinted with permission from [47], 2008, American Chemical Society.

I now turn to a more recent study of Andersson and colleagues, who used EPR to probe the heme iron environment in the cytochrome c of *Pseudimonas aeruginosa* (Pa *c_551_*) and *Nitrosomonas europaea* (Ne *c_552_*), which are both class I cytochrome c proteins. In addition to the respective wild types, several mutants were investigated, which were expected to affect the methionine ligand of the heme group. The two proteins are monomeric, with a high helical content and positive reduction potentials (Figure 7). Pa *c_551_* donates electrons to cytochrome *cd_1_* in nitrite and nitrate respiration, making its function analogous to that of the mitochondrial cytochrome c discussed thus far. Ne *c_552_* is an electron donor for cytochrome oxidase and a diheme peroxidase, whereas it accepts electrons from the tetraheme cytochrome *c*_554_. The mutant studies aimed to explore the influence of N64 and its neighbors on Pa *c_551_.* Both proteins differ regarding their respective position 50, i.e., G in Ne *c-552* and N in Pa *c_552_.* The authors’ mutation replaced the G with an N in the former and the N with a G in the later.

**Figure 8:**
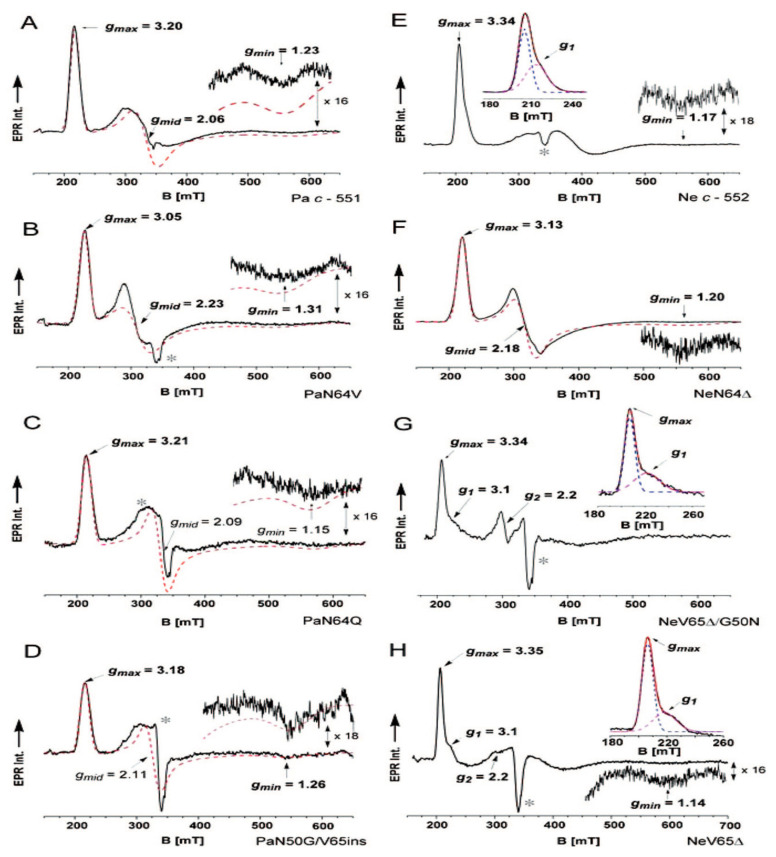
EPR spectra of (**A**) Pa *cyt_551_*, (**B**) *cyt_551_* N64V, (**C**) Pa *cyt_551_* N64Q, (**D**) Pa *cyt_551_* N50G/V65ins, (**E**) Ne *cyt_551_* cloned, (**F**) Ne *cyt_551_* N64Δ, (**G**) *cyt_551_* NeG50N/V65Δ and (**H**) Ne *cyt_551_* V65Δ in 50 mM HEPES buffer (pH 7.5), recorded at 10.0 K. The dashed lines represent simulated EPR envelopes. The asterisk indicates the Cu^2+^ signal (g ~ 2) resulting from an impurity. Experimental conditions can be inferred from the paper of Zoppellaro et al., from which this figure was reprinted with permission from [47], 2008, American Chemical Society.

The EPR spectra of these cytochromes (Figure 8) have in common that they all indicate three g-values diagnostic of a metal environment that is subject to rhombic deformations. Figure 4 depicts the energy scheme according to which the data were interpreted. It illustrates the lowering of the symmetry from octahedral to tetragonal and then to rhombic (D_4h_). The axial strain in the tetragonal field splits the lower-lying *t_2g_*-orbital into the higher-lying *d_xz_*/*d_yz_*- and the lower *d_xy_*-orbital by an energy Δ. With regard to the rhombic deformation that lifts the degeneracy between *d_yz_* and *d_xz_* (interaction energy V), the authors followed Walker [53] in that they distinguished between two types of heme–ligand complexes. Spectra with a large g_max_ signal (type I) are diagnostic of highly anisotropic low-spin hemes (HALS: highly axial low-spin). Type II depicts g_max_ values below 3.2, a small anisotropy and a more significant rhombic deformation (cf. the V/Δ values in Figure 4). The V/Δ value derived from the g-values of Pa *c-551* is 0.37, which puts it closer to type II than type I. For the respective N64V mutant, the ratio is 0.55, which is closer to the type II realm, thus indicating a stronger rhombic field. N interacts with the methionine 61 ligand. This interaction is abrogated in the mutant. Ne *c-552* has a V/Δ value of 0.24, which assigns it to type I. The deletion of N64 significantly increases this ratio to 0.5, moving it closer to type II. Interestingly, a replacement of the adjacent valine does not cause any significant changes in the energy ratio. These values should be compared with the ratio of 0.58 obtained for horse heart cytochrome c, which makes it a clear type II protein with strong rhombic deformation. A G50N replacement with V65 deletion for Ne *c-552* keeps the protein in the type I realm (V/Δ = 0.23). A N50G replacement combined with a V65 insertion is also inconsequential regarding the V/Δ-value. Interestingly, the authors could not obtain any correlation between the changes in the V/Δ and the orientation of M61.

A subsequent study of this group mutations of the *Hydrogenhobacter thermophilus cytochrome c_552_* and *Pseudomonas aeruginosa c_551_* aimed to explore the influence of mutations in the respective CxxCH region on the EPR spectrum [84]. These mutations were known to change the ruffling deformation of the heme. The results did not reveal any clear correlation between ruffling and V/Δ. The wild types and their mutants were all found to lie closer to the type I region, with minimal variations in its value.

**Figure 9:**
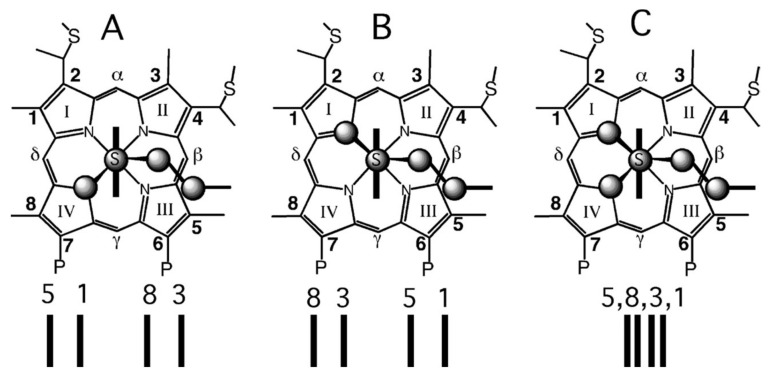
Schematic representation of heme axial M orientations (ball and stick format) and corresponding heme methyl 1H NMR chemical shift patterns observed in cytochromes c. The plane of the axial H-residue is shown as a bold black stick. (**A**) M orientation in PA *cyt_551_* (R), (**B**) M orientation in mitochondrial cytochromes c (S), (**C**) illustration of M fluxionality with M sampling, with the conformations shown in panels (**A**) and (**B**). The sticks below the structures represent the respective methyl shift pattern. Reprinted with permission from [47], 2008, American Chemical Society.

**Figure 10:**
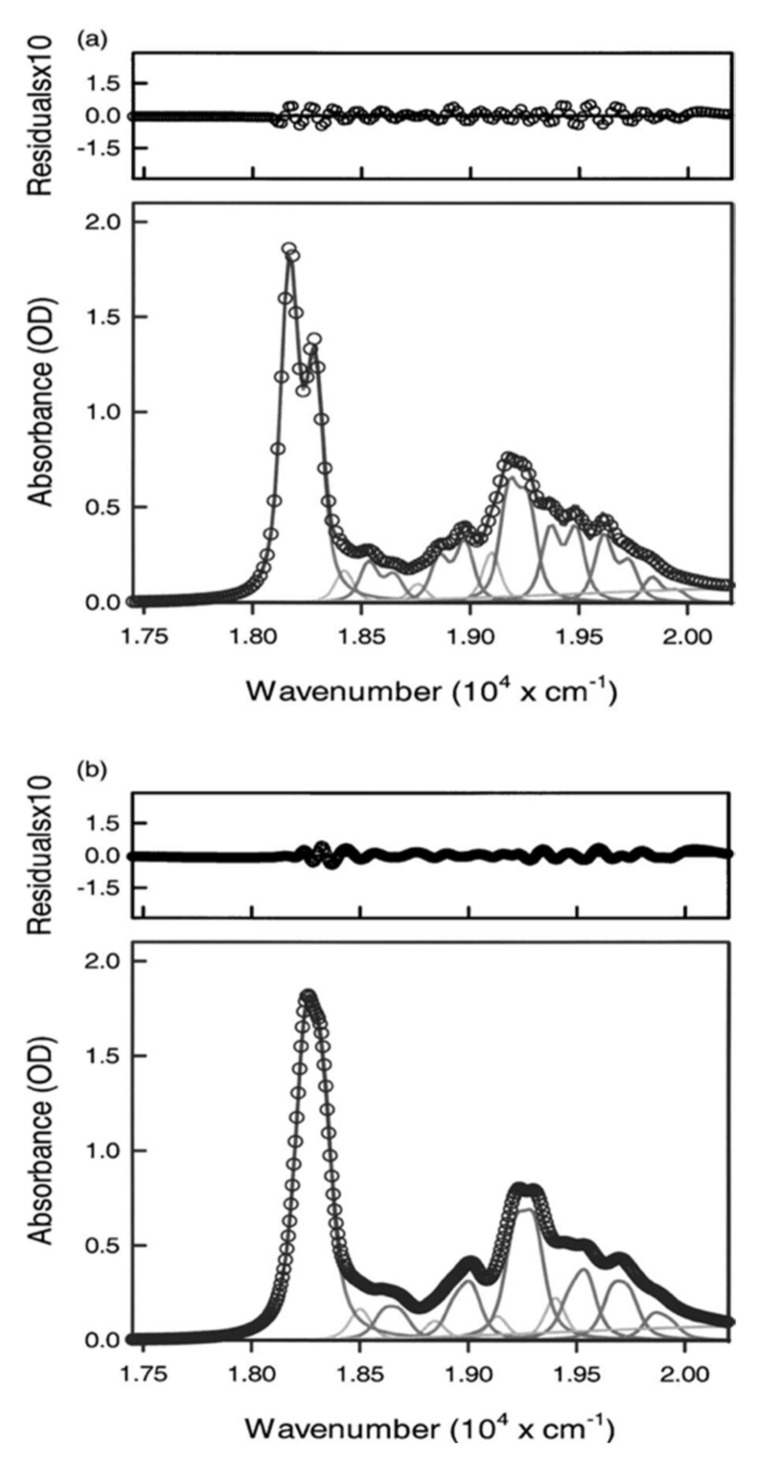
Q- and Qv-band region of the absorption spectra of horse heart (**a**) and yeast ferrocytochrome c (**b**) recorded at 10 K in a water-glycerol mixture. The spectral decomposition takes into account the observed splitting of vibronic transitions. The vibronic side band contributions can be assigned to vibrations of the heme macrocyle. The residual displayed on top of both figures reflects the quality of the performed fitting. Taken from Levantino et al. with permission [88], 2005, AIP Publishing.

Taken together, all the EPR studies discussed above are indicative of a rhombic heme environment. The low-spin character of the electron configuration puts the unpaired electron into the higher-lying *d_π_*-orbital, which, according to Zoppellaro et al., is *d_yz_* [47]. As discussed below, ruffling deformations cause an upshift of *d_xy_*, but in class I cytochromes, this change does not cause it to become the higher-lying orbital. Only in the presence of very strong π-electron-accepting ligands can the *d_xy_*-orbital be destabilized to such an extent [53]. While the rhombicity of low-spin ferric complexes has been well established by multiple EPR experiments, its origin and influences on functional properties (ligand binding, electron transfer) is rarely discussed in the literature. As pointed out by Walker, the symmetry of the heme depends on the orientation of the two axial ligands (Figure 9) [53]. If they are both planar and oriented parallel to each other, the effective symmetry is D_2h_. If they are perpendicular to each other, the symmetry is D_2d_. The methionine side chain is not truly planar, but its SH bond lines up with one of the Fe-N lines in both the R and the S configurations (Figure 9). This would correspond to a D_2d_ symmetry. The latter case of symmetry lowering involves a perturbation of B_1u_ symmetry, which eliminates the inversion center and corresponds to a ruffling deformation. This would not cause the rhombic environment of the heme iron. A much better candidate is static JT coupling (vide infra). The prime candidate is the low-wavenumber mode ν_24_ of *B_1g_* symmetry (Figure 6), which has been found at 228 cm^−1^ in the resonance Raman spectrum of horse heart ferri-cytochrome c [40]. This mode involves out-of-phase stretching vibrations of the N-C_α_ bond, with significant amplitudes of the four nitrogens (Figure 6). This mode would be ideal for lifting the degeneracy of the mixed (*3e_g_* − *d_π_*) and (*d_π_* − *3e_g_*) orbitals. It would cause a rhombic deformation along the normal coordinate of ν_24_ and produce a ligand field that has a *B_1g_* component. This symmetry-lowering effect would be present even if there were no other protein-induced heme perturbations. This is obviously not the case. In principle, the projection of the methionine ligand in both R and S onto the heme (Figure 10) lowers its symmetry to C_s_ by means of *B_1g_* and *E_u_* perturbations [85]. As shown below, the two thioether bridges of heme c are instrumental in imposing a ruffling deformation onto the heme group, but it is likely that they produce in-plane deformations as well (vide infra).

## 4. Absorption, Circular Dichroism and Resonance Raman Dispersion Spectroscopy

The resonance Raman, absorption and circular dichroism spectra of chromophores are interrelated, since the underlying physical processes all involve vibronic coupling, i.e., the change in the electronic Hamiltonian by nuclear vibrations along normal coordinates. For porphyrins, this leads to a breakdown of the Born–Oppenheimer approximation, which particularly affects resonance Raman scattering in the Q-band region due to complex multimode mixing effects [48,69,86]. The canonical adiabatic Albrecht theory of resonance Raman scattering, though widely popular, insufficiently accounts for the resonance excitation profiles in the Q-band region [69].

Detailed theoretical descriptions of the relationship between absorption, circular dichroism and resonance Raman scattering have been provided in earlier review articles, to which the interested reader is herewith referred [63,87]. Here, I confine myself to a more qualitative discussion. As mentioned earlier, the optical absorption spectra of both redox states of mitochondrial cytochrome c can be understood in terms of electronic transitions from the ground state into the excited electronic states of Q and B (Equations (6) and (10)). The lower-lying Q-state transition is much less intense due to the intensity borrowing of the B-state from the Q-state transition by a configuration interaction. If the two ground-state orbitals *3a_1u_* and *3a_2u_* were to be accidentally degenerate, the effective oscillator strength of the Q-band would be zero or very weak. Its apparent integrated molar absorptivity is a measure of de-mixing, where the *ν*-parameter in Equation (10) departs from π/4 [62]. For ferrocytochrome c, it is approximately 0.13 [48]. The so-called Q_v_-band, which is clearly resolved on the high-energy side of the Q_0_-band in the spectrum of ferrocytochrome c, stems predominantly from vibronic coupling between the Q- and B-states [88]. In the first order, this can be accounted for by time-independent Rayleigh–Schrödinger perturbation theory which leads to:

(17) $$\left|Q_{j},1_{k}\rangle =|Q_{j0},1_{k}\rangle +\frac{\left\langle B_{i},0_{k}|\frac{\partial {\hat{H}}_{el}}{\partial q_{k}^{\Gamma }}\cdot q_{k}^{\Gamma }|Q_{j},1_{k}\right\rangle }{E_{B_{i}}-E_{Q_{j}}-\Omega_{k}^{Q}}|B_{i},0_{k}\right\rangle$$

This is the so-called Herzberg Teller expansion. It describes the mixing of the first excited state of the *k*-*th* vibration in the excited Q-states with the respective vibrational ground state of the excited electronic B-states. The subscripts *i* and *j* represent x and y. *E_Bi_* and *E_Qj_* are the eigenenergies of the B_i_- and Q_j_-states in the unit of cm^−1^ in the absence of vibronic coupling. The vibronic coupling operator has already been introduced in Equation (15). $\Omega_{k}^{Q}$ is the wavenumber of the *kth* vibrational mode in the excited Q-state, which is generally not identical to the wavenumber in the electronic ground state [48,69,75]. The numbers 1 and 0 in the bras denote vibrational quantum numbers. It should be noted that, for the sake of brevity, coupling with the higher-lying vibrational B-states has been omitted in Equation (17).

Herzberg–Teller coupling, as described in Equation (17), transfers oscillator strength from the $\left|g,0_{k}\rangle \to \right|B_{i},0_{k}\rangle$ to the $\left|g,0_{k}\rangle \to \right|B_{i},1_{k}\rangle$ transition, which, in spite of the large energy difference between the Q- and B-states, becomes significant owing to the large oscillator strength underlying the B-band transition. In D_4h_ symmetry, all the in-plane modes except for the *E_u_*-modes can contribute to the vibronic side band of the Q transition. As shown by Levantino et al., the dominant contributions arise from high-wavenumber *A_2g_*-type modes owing to their large vibronic coupling strength [88].

In principle, Herzberg–Teller coupling also contributes to transitions into the $\left|B_{i},1_{k}\right\rangle$ states, but intensity borrowing from $\left|Q_{i},0_{k}\right\rangle$ is insignificant because of the weak oscillator strength of the Q-band transitions. The (not spectroscopically) resolved vibronic side band of the B-band transition is governed by intrastate Franck–Condon and, to a lesser extent, by Jahn–Teller coupling [67,86,89]. While the former requires *A_1g_* symmetry in D_4h_, the latter involves modes of *B_1g_* and *B_2g_* symmetry. If the B-state displacement along the respective normal modes is small (as it is for large aromatic ring systems), one can again employ Raleigh–Schrödinger perturbation theory to obtain:

(18) $$\left|B_{i},1_{k}\rangle =\right|B_{i},1_{k}\rangle_{0}+\frac{1}{\Omega_{k}^{B}}\langle B_{i}\left|\partial H_{el}/\partial q_{k}^{\Gamma }\right|B_{j}\rangle \cdot \left(\left|0_{k}^{g}\rangle \langle 0_{k}^{g}\right|q_{k}^{\Gamma }\left|1_{k}\rangle -\right|2_{k}^{g}\rangle \langle 2_{k}^{g}\left|q_{k}^{\Gamma }\right|1_{k}^{g}\rangle \right)$$

This approach follows Garozzo and Galluzi in that it keeps the vibrational function of the ground state and treats the displacement of the excited state along the coordinate *q_k_* as intrastate coupling between B-state components [90]. As shown earlier, the *A_1g_*-, *B_1g_*- and *B_2g_*-modes can contribute to the vibronic coupling matrix element in Equation (18). For A_1g_, this is Franck–Condon-type coupling, whereas the *B_1g_*- and *B_2g_*-modes produce Jahn–Teller coupling. A similar approach can be used for the Q-band. However, owing to the weak transition dipole moment of the corresponding transition, the respective contributions to the Q_v_-band are weak. The situation is quite different for resonance Raman scattering in the Q-band region (vide infra) [69,91].

How do symmetry-lowering perturbations affect the absorption spectrum? Owing to large width of the spectroscopic bands, the spectral resolution is low, which makes the identification of these perturbations difficult. For ferrocytochrome c, this problem can be circumvented by measuring the Q-band absorption at low temperatures (10 K) in glycerol/water mixtures [88]. The corresponding spectra of horse heart and yeast cytochrome c are shown in Figure 10. The Q_0_-band of horse heart cytochrome c is visibly split. Different vibronic contributions (Equation (17)) to the Q_v_-band are thus resolved, and identifiable components can be reproduced by doublets of Voigtian bands. The splitting is less pronounced for yeast cytochrome c, but its existence is still inferable from the asymmetric shape of the Q_0_-band (Figure 10, lower panel). The spectral analysis of Levantino et al. yielded Q_0_-band splittings of 116 and 77 cm^−1^ for the horse heart and yeast cytochrome c, respectively [88]. Earlier measurements of Q-band splitting yielded values between 100 and 120 cm^−1^ for other reduced mitochondrial cytochrome c derivatives (tuna, chicken, bovine, porcine). This splitting is clearly diagnostic of removal of the degeneracy of Q_0x_ and Q_0y_. The asymmetry of the band profile reflects the different transition dipole moments of the two components.

In an earlier study, Manas et al. attributed the splitting to the influence of the (rather strong) electric field that the protein and the peripheral substituents produce in the heme plane [92]. The first-order effect of an electric field whose vector does not exactly coincide with a C_m_-Fe-C_m_ line causes different blueshifts of the eigenenergies of Q_x_^0^ and Q_y_^0^ by means of a quadratic Stark effect and, thus, the removal of the *x,y* degeneracy. However, while this effect is significant for the B-state (vide infra), owing to the large transition dipole moment, it is insufficient for reproducing the Q-band splitting. Based on electrostatic calculations, Manas et al. suggested that the splitting is instead caused by the quadrupole moment of the electric field. As argued by Levantino et al., the coupling matrix element accounting for such an effect can be described as the intrastate electronic coupling of the type $<Q_{x}^{0}\left|V^{B_{1g}}\right|Q_{x}^{0}\rangle =-<Q_{y}^{0}\left|V^{B_{1g}}\right|Q_{y}^{0}\rangle$, where $V^{B_{1g}}$ is an electronic perturbation potential of the type introduced in Equation (7) [88]. While this type of electronic perturbation can split the Q-state, it does not account for the different transition dipole moments of $\left|Q_{x}\right\rangle$ and $\left|Q_{y}\right\rangle$. To arrive at a consistent description of band splitting and asymmetry, Levantino et al. considered interstate coupling in the presence of asymmetric perturbations. This led them to the following set of equations for the eigenenergies in second order:

(19) $$E_{Qx}=E_{Q}^{0}+\frac{{\left(V_{Q_{x}B_{x}}^{A_{1g}}+V_{Q_{x}B_{x}}^{B1_{g}}\right)}^{2}+{\left(V_{Q_{x}B_{y}}^{B_{2g}}+V_{Q_{x}B_{y}}^{A_{2g}}\right)}^{2}}{E_{Q}^{0}-E_{B}^{0}}$$

(20) $$E_{Qy}=E_{Q}^{0}+\frac{{\left(V_{Q_{y}B_{y}}^{A_{1g}}-V_{Q_{y}B_{x}}^{B1_{g}}\right)}^{2}+{\left(V_{Q_{y}B_{x}}^{B_{2g}}-V_{Q_{y}B_{x}}^{A_{2g}}\right)}^{2}}{E_{Q}^{0}-E_{B}^{0}}$$

Equations (19) and (20) implicate that Q-band splitting requires the simultaneous presence of *A_1g_* and *B_1g_* and/or *A_2g_* and *B_2g_* perturbations. In the framework of the four-orbital model, intrastate and interstate electronic coupling are not independent (cf. Equation (10)).

Levantino et al. found that, for horse heart cytochrome c, the $V_{Q_{i}Q_{i}}^{B_{1g}}$ value that emerged from their analysis of the Q-band profile was very close to the one derived from an estimation of quadrupole coupling by Manas et al. This led them to the conclusion that the Q-band splitting does indeed predominantly result from the quadrupole moment of the electric field in the heme plane, which they estimated to be predominantly produced by the charges on the two ligands and the propionic acid substituents.

In addition to the electronic contributions described above, a quantitative reproduction of the Q-band profiles in Figure 10 requires the consideration of vibronic perturbations, which are briefly described as follows. As shown by Zgierski and colleagues, vibronic perturbations can be accounted for by using the Hamiltonian in Equation (7) for the vibronic coupling operator [16,68,93]:

(21) $$\frac{\partial {\hat{H}}_{el}}{\partial q_{k}}=\frac{\partial {\hat{H}}_{el,0}}{\partial q_{k}}+\sum_{\Gamma }\frac{\partial {\hat{V}}^{\Gamma }}{\partial q_{k}}$$

While the first term of the r.h.s. transforms as the representation of *q_k_*, the second (vibronic perturbation) term transforms as the product *Γ_k_* × *Γ.* Hence, the symmetry of the vibronic coupling operators reads as a sum of D_4h_ symmetry representations. To illustrate the significance of vibronic perturbations, let us consider a *B_1g_*-mode. This could contribute to Jahn–Teller coupling between the Q- and B-states, respectively. It would be accounted for by the matrix element in Equation (18), which would transform in the manner of *B_1g_.* In the presence of *B_1g_*-type perturbations (vide supra), *B_1g_* × *B_1g_* = *A_1g_* symmetry would be admixed with the coupling operator, which would add Franck–Condon activity to the considered mode *q_k_.*

**Figure 11:**
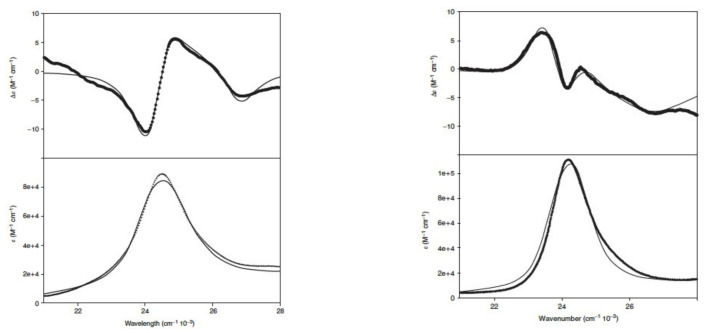
Circular dichroism (**upper** figures) and Soret band absorption (**lower** figures) of oxidized (**left**) and reduced horse heart cytochrome c (**right**). The solid lines result from a fit of a vibronic coupling model with the corresponding band profiles. Details of the theory can be inferred from [50]. Reprinted with permission from [50], 2008, American Chemical Society.

The significance of vibronic perturbations can best be illustrated by a closer look at the visible circular dichroism spectra. Figure 11 (left) depicts the CD and absorption spectra of oxidized horse heart cytochrome c in the B-band (Soret band) region. The CD profile can be described as a negative couplet. In the framework of the four-orbital model, its very existence is indicative of a split between *B_x_* and *B_y_*, with opposite rotational strengths of the respective electronic transitions [49]. The corresponding spectra of reduced cytochromes are slightly more complex (Figure 11, right). Schweitzer-Stenner used a vibronic coupling model to reproduce the shape of three couplets and the corresponding absorption bands depicted for three different mitochondrial cytochrome cs [50]. The author found that while the contribution of vibronic perturbations to the B-band splitting is small (41 cm^−1^ added to 505 cm^−1^ for horse heart and cow and 12 cm^−1^ to 380 cm^−1^ for yeast), the situation is quite different for ferricytochrome c, where the vibronic perturbations add 390 cm^−1^ to 126 cm^−1^ for the horse heart, 127 cm^−1^ to 186 cm^−1^ for cow, and 25 cm^−1^ to 217 cm^−1^ for yeast. While B_x_ lies at lower energies than B_y_ in the oxidized proteins, it is at higher energies in ferrocytochrome c. Vibronic perturbations have an even more pronounced influence on the vibronic side bands of B_x_ and B_y_. The underlying reason is reflected in the vibronic coupling matrix elements:

(22) $$\left\langle B_{x}|\frac{\partial {\hat{H}}_{el}}{\partial q_{k}}|B_{x}\rangle =\langle B_{x}|\frac{\partial {\hat{H}}_{el,0}}{\partial q_{k}^{A_{1g}}}+\frac{\partial {\hat{V}}_{el}^{B_{1}g}}{\partial q_{k}^{A_{1g}}}|B_{x}\right\rangle \;\left\langle B_{y}|\frac{\partial {\hat{H}}_{el}}{\partial q_{k}}|B_{y}\rangle =\langle B_{y}|\frac{\partial {\hat{H}}_{el,0}}{\partial q_{k}^{A_{1g}}}-\frac{\partial {\hat{V}}_{el}^{B_{1}g}}{\partial q_{k}^{A_{1g}}}|B_{y}\right\rangle$$

where *B_1g_*-type vibronic perturbations add coupling strength to one side band and reduce it for the other one. As a consequence, the vibronic side bands of B_x_- and B_y_ can appear rather different in the presence of the *B_1g_* perturbations of the heme group. It should be noted, in the context, that this influence of vibronic perturbations on the shape of the absorption bands was discussed more than 30 years ago by Bersuker and Stavrov [94]. Their contribution was mostly ignored in the porphyrin/heme protein literature. More recently, Schweitzer-Stenner et al. [65] invoked the above vibronic coupling scheme to explain the dispersion of the ratio of polarized absorption observed for the single crystals of myoglobin cyanide by Eaton and Hochstrasser [95].

**Figure 12:**
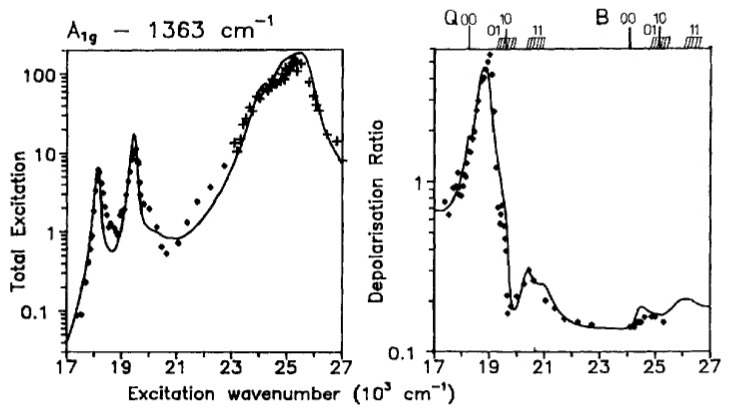
Depolarization ratio dispersion (**left**) and resonance excitation profiles (**right**) of the oxidation marker *A_1g_*-type mode ν_4_ of horse heart ferrocytochrome c. The solid lines in the figures result from fits of a vibronic coupling model that considers symmetry-lowering perturbations and multimode mixing. Details can be inferred from [48]. Resonance positions of Raman excitation are indicated at the top of the figures with regard to the occupation of vibrational states. The figure was reprinted with permission from reference [48], 1991, Wiley & Sons.

I finish this chapter with a discussion of how electronic and vibronic perturbations affect resonance Raman scattering. As mentioned above, absorption and resonance Raman scattering are interrelated in that the Franck–Condon, Jahn–Teller and Herzberg–Teller active modes are also resonance-Raman-active. On the most elementary level, this can be explained by inserting the expansion in Equations (17) and (18) into the Kramers–Heisenberg–Dirac equation. The interested reader can infer details from earlier review articles [63]. In an ideal D_4h_ symmetry, the modes of *A_1g_*, *B_1g_*, *B_2g_* and *A_2g_* are resonance-Raman-active. While bands assignable to the *A_1g_*-modes dominate spectra taken with B-band excitation, non-symmetric and antisymmetric modes dominate spectra taken in the Q-band region. In D_4h_ symmetry, these modes can be identified by their depolarization ratios, i.e., 0.125 for *A_1g_*-modes, 0.75 for *B_1g_* and *B_2g_* and infinite for *A_2g_*-modes, independent of the excitation wavelength [77,96]. The experimental values were indeed found to be close to these expectation values for Ni(II) porphine. The situation is completely different for cytochrome c. Figure 12 shows the depolarization ratio and the normalized scattering intensity of the ν_4_-mode of horse heart ferrocytochrome c as a function of the excitation wavenumber. While the value of 0.14 in the pre-resonance region of the B-band is close to the D_4h_-value, it increases towards the Q-band region to rather high values of ca. 20. In other words, the band becomes inverse-polarized between the respective Q_0_ and Q_1_ position, where the data exhibit local minima. The solid lines in the two figures result from a self-consistent fit, which is described in detail in [48]. The depolarization dispersion could only be reproduced by a vibronic coupling mixture of *A_1g_*, *B_1g_* and *A_2g_* symmetry. Schweitzer-Stenner et al. interpreted the result as indicative of symmetry-lowering vibronic perturbations of *B_1g_* and *A_2g_* symmetry. An analysis of the depolarization ratio and excitation profiles of other Raman active modes led to a similar conclusion. Tthe validity of these data was questioned by Hu et al. based on a comparison of the polarized Raman spectra of isotopically substituted hemes in ferrocytochrome c [40]. The spectra were measured at cryogenic temperatures. The authors claimed that the reported deviations of depolarization ratios from the D_4h_ expectation values solely reflect the overlap of the bands originating from the macrocycle and peripheral substituents. In response to Hu et al., Schweitzer-Stenner performed a more detailed spectral analysis of ferrocytochrome spectra, which reproduced the previously reported depolarization dispersion of ν_4_ [97].

Contrary to the oxidized cytochrome c case, there cannot be any JT-induced distortion in the reduced state owing to the ^1^*A_1g_*-type electronic ground state. However, the occurrence of *B_1g_*-deformation can easily be understood as being caused by the two axial ligands (vide supra). While the depolarization ratio of ν_4_ clearly reveals an antisymmetric contribution to its Raman tensor, the deformations of *A_2g_* symmetry by a vibronic perturbation of the same symmetry is unlikely to be dominant because of the comparatively high wavenumber of the lowest *A_2g_*-mode (243 cm^−1^, compared with 168 cm^−1^ (*B_1g_*) and 144 cm^−1^ (*B_2g_*) for Ni(II)-octaethylporphyrine). A much more sensible interpretation can be provided if one invokes a combination of out-of-plane deformations based on the group theoretical reasoning below. To this end, I rewrite Equation (21) as follows:

(23) $$\frac{\partial {\hat{H}}_{el}}{\partial q_{k}}=\frac{\partial {\hat{H}}_{el,0}}{\partial q_{k}}+\sum_{\Gamma }\frac{\partial V^{\Gamma }}{\partial q_{k}}=\frac{\partial {\hat{H}}_{el,0}}{\partial q_{k}}+\sum_{l}\frac{\partial^{2}{\hat{H}}_{el,0}}{\partial q_{k}\partial q_{l}^{\Gamma }}\cdot \delta Q_{l}^{\Gamma }+\frac{1}{2}\sum_{l}\sum_{m}\frac{\partial^{3}{\hat{H}}_{el,0}}{\partial q_{k}\cdot \partial q_{l}^{\Gamma }\cdot \partial q_{m}^{\Gamma^{\prime }}}\cdot \delta Q_{l}^{\Gamma }\cdot \delta Q_{m}^{\Gamma^{\prime }}$$

where the second term in Equation (21) is replaced by a second-order Taylor expansion of the vibronic coupling operator with respect to the normal coordinate deformations *δQ*. The second derivative of the expansion transforms in the manner of the product representation *Γ_k_* × *Γ_l_*_._ It accounts for the contribution of all the in-plane deformations to the vibronic coupling operator. The third derivative transforms in the manner of the triple-product representation *Γ_k_* × *Γ_l_* × *Γ_m_′.* It can be used to describe the influence of the combination of two out-of-plane deformations. As discussed in more detail below, the dominant out-of-plane deformations of the heme group in cytochrome c are ruffling and saddling (Figure 5). Since the corresponding symmetries are *B_1u_* and *B_2u_*, the product symmetry for an *A_1g_*-mode would be *A_2g_.* The assignment of the antisymmetric contribution to the Raman tensor of ν_4_ to this combination of out-of-plane deformations is consistent with the occurrence of a similarly large antisymmetric contribution to the ν_4_-mode of Ni-octaethyltetraphenyl-porphyrin [75], which is predominantly saddled, with some minor contributions of ruffling deformations [98] and with far less pronounced contributions in the case of other heme proteins, such as myoglobin and hemoglobin [71].

**Figure 13:**
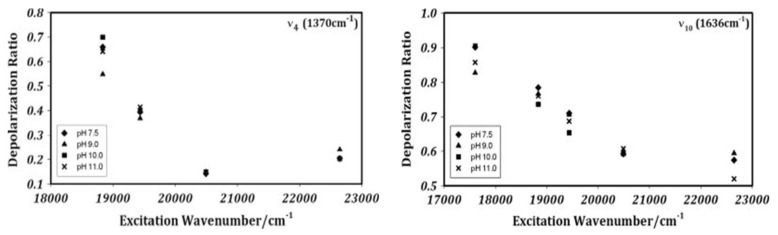
Depolarization ratio dispersion of the oxidation marker ν_4_ (**left**) and the spin marker ν_10_ (**right**) of horse heart ferricytochrome c in the pre-resonance regions of the *Q_v_*- and B-bands obtained at the indicated pH values. Reprinted with permission from [51], 2001, Wiley & sons.

As stated above, the heme of reduced cytochrome c cannot be subject to JT distortions of the ground state. Since such a distortion is supposed to exist in ferricytochrome c, one would expect a more pronounced deviation from the D_4h_-values of the depolarization ratio, particularly with excitation wavelengths in the pre-resonance and resonance regions of the B-band. Figure 13 exhibits the depolarization ratios of ν_4_ (*A_1g_*, 0.125) and ν_10_ (*B_1g_*, 0.75) of oxidized horse heart cytochrome c as a function of the excitation wavenumber in the region between Q and B. The data cover the resonance region of the respective *Q_v_* excitation (at 19,500 cm^−1^). With 442 nm (22,624 cm^−1^) excitation, the depolarization for ν_4_ and ν*_10_* are 0.2 and 0.58, well above and below their respective D_4h_-values, respectively. As shown by Alessi et al., these values are diagnostic of the substantial *B_1g_*-deformation of the heme macrocycle, which by far exceeds the one in ferrocytochrome c [51]. Deformations of this symmetry add *B_1g_* symmetry to the Raman tensor of ν_4_ via the second term in the Taylor expansion of Equation (23). For *B_1g_*-modes, however, the corresponding perturbation term transforms in the manner of *B_1g_* × *B_1g_* = *A_1g_*, which leads to a reduction in the depolarization ratio from the D_4h_-value of 0.75. It should be noted that a similar dominant *B_1g_*-deformation was obtained earlier for the low-spin ferric state of myoglobin cyanide, which also has an *^2^E_g_* ground state, but not for the other myoglobin derivatives [65].

Taken together, the combined analysis of (low-temperature) optical absorption, B-Band circular dichroism and the resonance Raman data unambiguously shows that the heme macrocycle of ferri- and ferrocytochrome c is subject to in-plane *B_1g_* (rhombic) and out-of-plane *B_1u_* (ruffling) and *B_2u_* (saddling) deformations. In line with the results of EPR studies (vide supra), the *B_1g_* deformation in the oxidized protein, which can be predominantly ascribed to a rhombic distortion along the *ν_24_* coordinate, lifts the degeneracy of the d_π_ orbitals, as well as that of the *3e_g_* orbitals, of the heme macrocycle. The mixture of the two orbitals confers spin density onto the heme macrocycle and produces an *^2^E_g_* ground state of the heme group (macrocycle + iron). Additional symmetry-lowering deformations of the heme group in both redox states are conferred onto the heme by electronic perturbations produced by the charges on the two ligands and the two propionic acid charges Contrary to the assumption of Manas et al., it is not certain that both propionic acid substituents are protonated. The work of Bowler and coworkers [99] indicates that one of the two groups has exceptionally high pK values.

While this chapter focused on the deformations produced by the in-plane components of electronic and vibronic perturbations, out-of-plane deformations were briefly discussed after they were inferred from the antisymmetric contributions to the Raman tensor of the totally symmetric ν_4_-mode. They will be dealt with in greater detail in the subsequent sections of this paper.

## 5. Out-of-Plane Deformations Inferred from the Crystal Structures of Cytochrome c

Th exploration and determination of the (mostly) out-of-plane deformations of metal porphyrins in solution and in heme proteins have been pioneered by Jentzen and Shelnutt [58,70,71,97]. While work on model porphyrins indicated the functional relevance of these deformations, it took some time and the work of Bren and coworkers to show that they affect the redox potentials, electron transfer kinetics and ligand binding properties. In this section, I review the results of the structural analysis of cytochrome c derivatives. The work of Bren and coworkers is described in the subsequent section.

**Figure 14:**
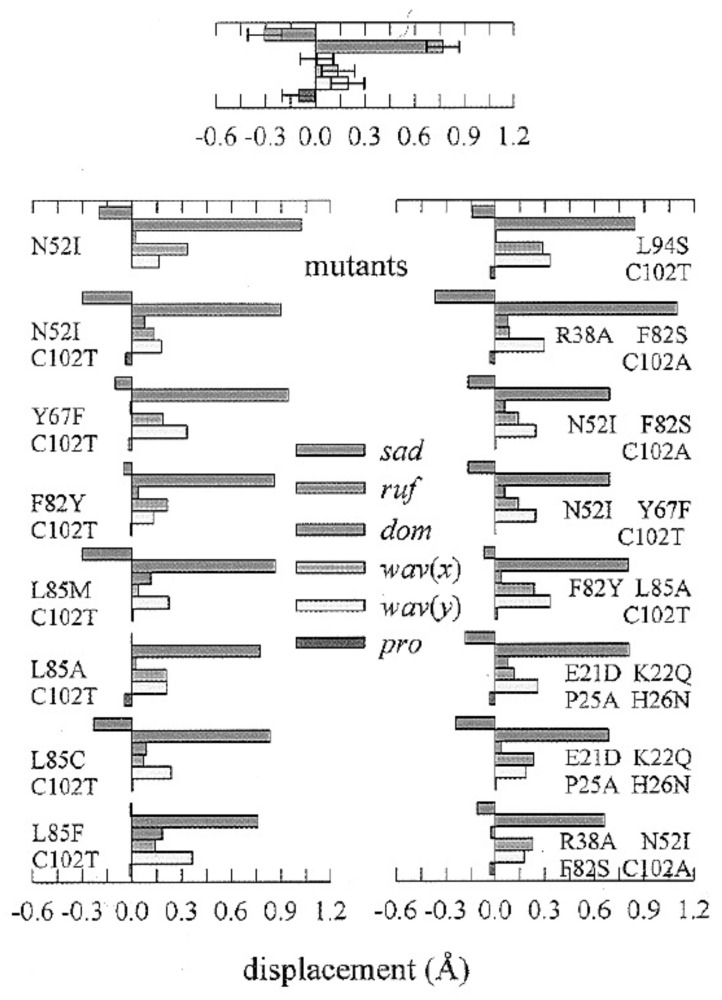
Bar diagram representing the out-of-plane deformations of the heme group in yeast ferrocytochrome and the indicated mutants. The diagram, at the top, exhibits the deformations of the C102T mutant, which is generally considered as the wild type. This mutation solely serves the purpose of avoiding disulfide bridging between proteins. The figure was taken from [70] and slightly modified, with permission from Ref. [70], 1997, American Chemical Society.

Equation (14) describes the total porphyrin deformation in which the contributions of all the symmetries are added up. Generally, Jentzen et al. distinguished between deformations of different symmetries. They showed that a minimal basis set of low-wavenumber modes is frequently sufficient to reproduce a certain type of deformation. Figure 14 shows the results of their analysis of yeast ferrocytochrome c and some of its mutants. Note that C102T yeast cytochrome c is generally considered as the wild type. The indicated mutation just prevents the formation of protein dimers via the disulfide bridges between C102 residues. Ruffling is the dominant deformation (0.8 Å), followed by a negative saddling deformation (−0.3 Å). Waving is small but still significant (0.2 Å). Note that it is the coexistence between ruffling and saddling that explains the antisymmetric contribution to the Raman tensor of the ν_4_-mode [48,51]. The investigated mutants are all in the heme pocket close to the M80 ligand, which can be expected to change the interactions between the residues (Y67F, N52I), as well as the interactions between the water molecules in the heme pocket [100]. However, in contrast to what one might expect, these substitutions have very limited influences on the distribution of the out-of-plane deformations. This result should be contrasted with the ones observed in the resonance Raman and low-temperature absorption studies of some of these mutants, which reveal substantial changes in in-plane deformations, particularly *B_1g_* deformations [101]. A different picture arises if one compares the out-of-plane deformations of different mitochondrial cytochrome c proteins (Figure 14, right column). Compared with horse heart cytochrome c, the reduced tuna cytochrome exhibits less ruffling and more saddling. A comparison of the mitochondrial cytochromes with the cytochrome c’ and cytochrome c_2_ derivatives reveals major differences. For three representatives of the former, particularly for cytochrome c’ of *R. molischianum*, Jentzen et al. observed a dominance of saddling deformation. Among the three investigated forms of cytochrome c_2_, only the heme group of *R. rubrum* depicts noticeable out-of-plane deformations (mostly ruffling). The authors also investigated the crystal structures of oxidized cytochrome c proteins and found them to be akin to the ones in the reduced state [71]. As shown above, this is obviously not true for in-plane deformations, which affect the oxidized protein more than the reduced one.

**Figure 15:**
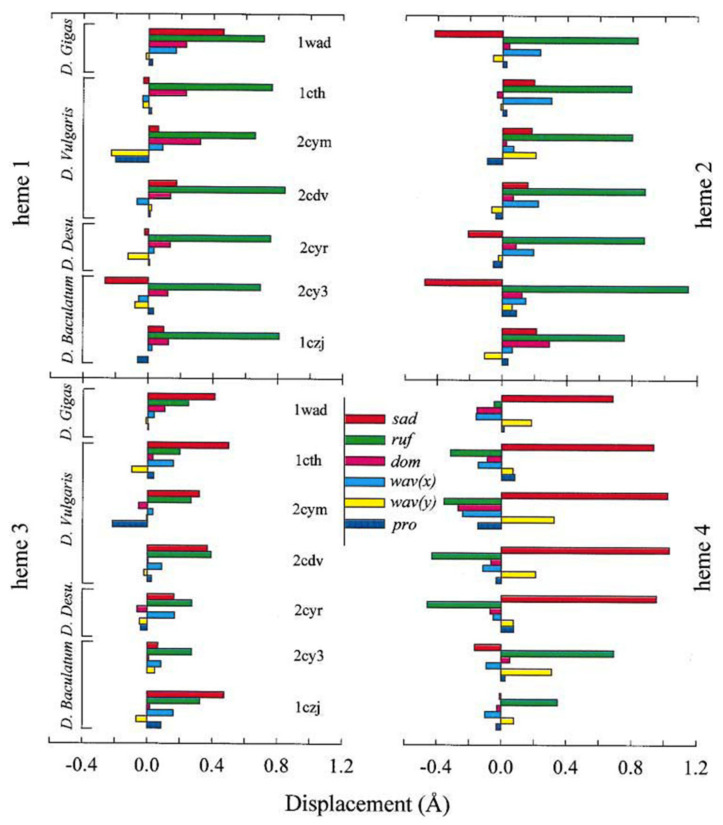
Bar diagrams representing the indicated out-of-plane deformations of the four heme groups of the indicated cytochrome c_3_ proteins. References to the respective X-ray structures and the papers in which they were published can be inferred from the caption of Figure 7 in Jentzen et al. [71], from which the figure was taken with permission, 1998, Elsevier.

Some light is shed on what might be deemed as the structural determinants of out-of-plane deformations by the out-of-plane deformations of the four heme groups of cytochrome c_3_ from *D. gigas*, *D. vulgaris*, *D. desulfuricans* and *Desulfomicrobium baculatum* (Figure 15). Hemes 1 and 2 are predominantly subject to ruffling. The contribution of saddling varies between the proteins. Contributions from doming and waving are minor but not insignificant. The picture is totally different for most of the investigated heme 3 and heme 4 chromophores, where saddling is the dominant out-of-plane deformation. An exception from the rule is the heme 4 of *Desulfomicrobium baculatum*, which is predominantly ruffled. Jentzen et al. explained the latter finding based on differences between the protein segments flanked by the two cysteins covalently linked to the heme group, which is of the CxxC type for heme 4 but encompasses four residues in heme 4 in the other investigated cytochrome c_3_ species. However, the picture provided by the entire dataset is equivocal in that hemes 1 and 3 both contain the classical CxxC segment of mitochondrial cytochrome c but exhibit rather different out-of-plane deformations. The contributions of the two thioether linkages and the amino acid residues between the two cysteins are illuminated in context of NMR studies on the various cytochrome c derivatives, which are described in the next section.

Differences between cytochrome c and other heme proteins are worth mentioning. Deoxyhemoglobin A is mostly subject to doming, which was originally described as iron out-of-plane displacement [71]. This deformation is reduced in the oxygenated state. In oxidized hemoglobin, the heme group is slightly distorted along nearly all the out-of-plane deformations. The hemes in peroxidases, such as the classical horseradish peroxidase, are dominantly saddled [71].

## 6. Out-of-Plane Deformations Probed by NMR Spectroscopy

Nowadays, NMR spectroscopy is generally employed using the two- and three-dimensional versions to probe the structures of proteins in solution. With regard to proteins such as cytochrome, NMR has been shown to compete with the canonical X-ray structure analysis, which relies on the availability of protein crystals [23,81]. Here, I focus (mostly) on the one-dimensional ^1^H and ^13^C NMR of heme groups in cytochrome c derivatives. The applicability of these techniques results from modifications of the chemical shifts owing to the presence of single-occupied molecular orbitals that Bren termed SOMO [59]. In the framework of the classical four-orbital model, where the heme and metal orbitals are well separated, the unpaired electron of a low-spin ferric heme resides solely in the *d_π_* or the *d_xy_* orbital. However, as described above, the mixing of *3e_g_* and *d_π_* orbitals confers spin density onto the porphyrin macrocycle in the electronic ground state. As shown below, ruffling deformations provide another mode of porphyrin–metal orbital mixing that can modify the spin density of the heme group.

As outlined by Bren [59], paramagnetic systems such as Fe^3+^-hemes give rise to a so-called hyperfine shift:

(24) $$\delta_{hf}=\delta_{obs}-\delta_{dia}$$

where $\delta_{obs}$ is the experimentally observed chemical shift of a nucleus interacting with an unpaired electron, while $\delta_{dia}$ is the corresponding shift expected of the diamagnetic system. The hyperfine shift is generally ascribed to three additive sources, i.e., the Fermi contact shift, the sign of which reflects the z-component of the spin of the SOMO electron (positive or negative), pseudocontact shift, which reflects paramagnetic anisotropy, and a ligand-centered pseudocontact shift. The pseudocontact shift is a rather convoluted function of the nuclear coordinates and the magnetic susceptibility tensor of the heme–ligand complex. Details can be found in the very enlightening paper of Banci et al. [54].

Before I discuss the relationship between out-of-plane deformations and chemical shifts, I would like to draw the reader’s attention to the influence of the axial ligands, in particular methionine. As mentioned above, the orientation of both the methionine and the imidazole side chains lowers the symmetry of the heme group by inducing in-plane deformations. In the oxidized state, a JT distortion along the ν_24_-coordinate must be added to the picture. The perturbation Hamiltonian in Equation (13) represents an interaction that causes the mixing of the two iron-porphyrin states, which can be described as a linear combination of the wavefunction in Equation (13):

(25) $$\left|\psi_{FeP,1}^{’}\rangle =cos\beta \cdot \left|d_{xz}+3e_{gx}\rangle +sin\beta \cdot \right|d_{yz}+e_{gy}\right\rangle \;\left|\psi_{FeP,2}^{’}\rangle =cos\beta \cdot \left|d_{yz}+3e_{gy}\rangle -sin\beta \cdot \right|d_{xz}+e_{gx}\right\rangle$$

where *β* is the angle of rotation of the *x*,*y* coordinate system, which diagonalizes the Hamiltonian in Equation (13). Equation (25) is equivalent to the one presented by Banci et al., with a slightly different notation [54]. Karplus and Fraenkel showed that the rational angle *β* determines not only the energy splitting between the x- and y-polarized states (that follows, of course, from elementary quantum mechanics) [102] but also the spin density distribution of the heme carbons. As delineated by Banci et al., the hyperfine shifts of protons and ^13^Cs are different for oxidized horse heart cytochrome c in that the signals of the methyl protons shift downfield, while those of carbon shift upfield. The temperature dependence of the paramagnetic contribution to the chemical shift generally does not obey Curie’s law in that it does not reach zero at an infinite temperature and exhibits positive as well as negative slopes.

**Figure 16:**
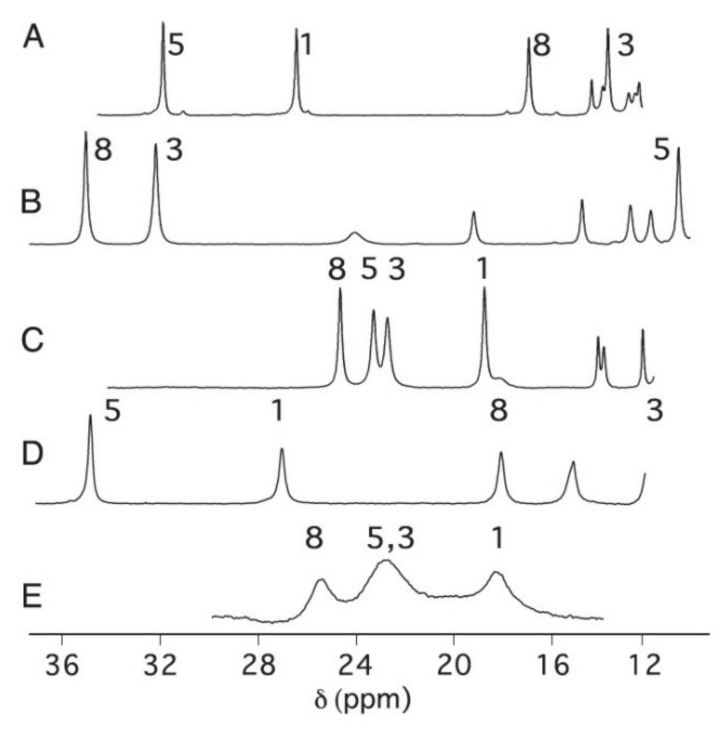
Downfield regions of the ^1^H NMR spectra of the following oxidized cytochromes: (**A**): *Pa cyt_551_* in aqueous solution, (**B**): horse heart cytochrome c, (**C**): *Ht cyt c_552_* in aqueous solution, (**D**): *Pa cyt_551_* in a solution containing 20 vol% CD_3_OD, (**E**): *Ht cyt_55s_* in a solution containing 20 col% CD_3_OD. Experimental details are provided in the paper of Zhong et al., from which the figure was taken [61] (open access). The numbering 5,3 represents two overlapping signals.

**Figure 17:**
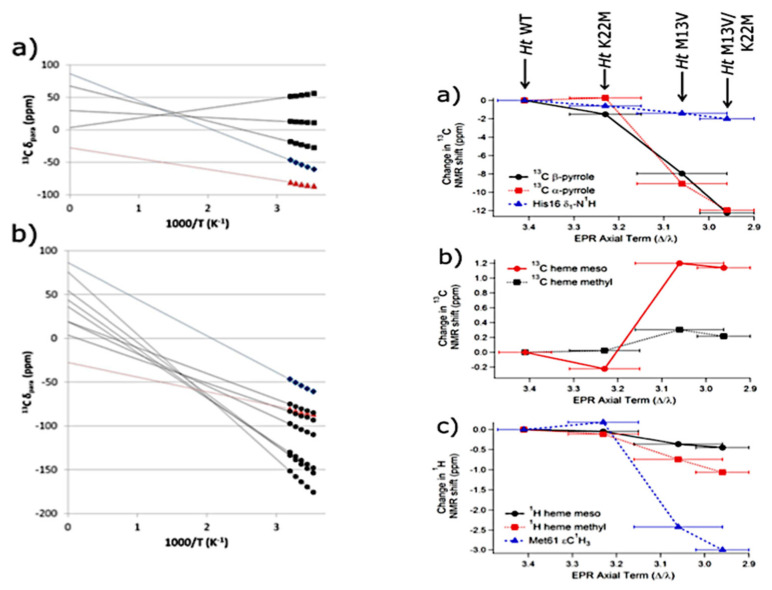
Left: (**a**) Temperature dependence of the three assigned β-pyrrole ^13^C shifts of *Pa cyt_551_* WT (black squares) compared with those of the two peaks with ambiguous assignments (blue diamonds, red triangles). (**b**) Temperature dependence of the seven assigned α-pyrrole ^13^C shifts of *Pa cyt_551_* WT (black circles) compared with those of the two peaks with ambiguous assignments (blue diamonds, red triangles). Right: Changes in the indicated chemical shifts caused by the indicated mutations of *Ht cyt_552_*, plotted as a function of the axial energy terms determined by EPR experiments. (**a**) ^13^C shifts of pyrrole carbons. The HH shift of the H17 side chain is shown for comparison. (**b**) ^13^C shifts of the methyl and *meso* carbons, (**c**) ^1^H shifts of *meso* and methyl groups compared with the shift of CH_3_ end group of M71. Reprinted with permission from [103], 2013, American Chemical Society.

Before entering a discussion on how out-of-plane deformations change the chemical shift of theme nuclei, the influence of the methionine ligand is briefly discussed. Figure 16 shows the downfield region of the ^1^H NMR spectra of five different ferric cytochrome c derivatives: *Pa-cyt_551_* (A), mitochrondrial horse heart cytochrome c (B), *Hydrogenobacter thermophilus (Ht) cyt_551_*, *Pa-cyt_551_* in a solution containing 20% CD_3_OD (D), and *Ht-cyt_551_*, also in a binary mixture of water and deuterated methanol. Here, I focus on a comparison of spectra A–C, which differ in the sequence of methyl proton signals. All these signals exhibit the same chemical shift in the absence of any electronic anisotropy. The differences between the spectra of A and B were assigned to two different orientations of the methionine side chain (termed A and B by Zhong et al. (Figure 17) [61]. The notation was later replaced by R and S (*vide supra*)). The spectrum of *Ht cyt_551_* is diagnostic of a dynamic equilibrium between R and S. Zhong et al. assigned the latter to the absence of hydrogen bonding involving the methionine side chain and structural effects caused by the Q64 side chain.

The influence of heme ruffling on heme group chemical shifts was investigated by Kleingardner et al. by employing the mutants of *PA cyt_551_* and *Ht cyt_551_* [103]. Regarding the former, they measured the NMR spectra of an F7A mutant whose mutations are known to increase the ruffling deformation without significantly affecting the deformation along the other out-of-plane coordinates. For *Ht cyt_551_*, they investigated the mutants M13V, K22M and the respective double mutant. All these mutations were shown to increase ruffling. Figure 17 (left) shows the Curie plots for the paramagnetic shifts of the isotopically labeled (^13^C) α- and β-carbons of wild-type *PA cyt_551_.* Most of the plots approach values above zero for T -> ∞, which is indicative of hyper-Curie behavior. It reflects the fact that higher-lying excited electronic states (i.e., (*d_xy_*)^1^(*dπ* − *eg*)^4^) can become populated at room temperature.

A comparison of the investigated wild types and mutants revealed the following picture. In the *PA cyt_551_* F7A mutant (increased ruffling), the average heme methyl shifts (H and ^13^C) were found to increase, while the shifts of the pyrrole carbons decreased. In *Ht cyt_551_*, the same mutation decreased the ruffling. As a consequence, the respective chemical shift exhibited the opposite behavior.

Figure 17 (right) shows the plots of the average ^13^C and H shifts of the *Ht cyt_551_* derivatives as a function of the decreasing axial energy term Δ/λ, inferred from the EPR experiments. Δ denotes the energy splitting between the *t_2g_*- and *e_g_*-orbitals caused by the lowering of the symmetry of the metal’s ligand field from octahedral to tetragonal, whereas λ denotes the (thus far not explicitly considered) spin–orbit coupling energy. Can et al. deduced from EPR experiments on the same protein that an increase in ruffling results in a decrease in Δ [84]. Hence, the abscissa in Figure 17 can be read as representing heme ruffling. The data depicted therein clearly show how the chemical shifts of low-spin oxidized hemes can be utilized to probe heme ruffling. Overall, the data demonstrate that ruffling significantly affects the unpaired spin distribution of the macrocycle, which is an indicator of mixing between heme and iron orbitals.

Details regarding the ways in which ruffling changes the spin delocalization over pyrrole carbons, methyl carbons and methyl protons can be inferred from the very thorough study of Kleingardner et al. [101]. The authors also provided a detailed deconstruction of paramagnetic shifts into individual contributions. Here, I focus on how ruffling changes the mixing between heme iron and porphyrin orbitals. In the chosen D_4h_ reference system, ruffling transforms as *B_1u_.* Thus, on the orbital level, ruffling can mix the *d_xy_*-orbital (*B_1g_*) of the metal with the *3a_2u_*-orbital of the macrocycle, since *A_2u_* × *B_1g_* = *B_1u_*. Taking this orbital mixing into account, one must augment the set of electronic states described in Equation (5). Here, one must take into account the fact that, contrary to the mixing of the *d_π_*- and *3a_2u_*-orbitals, the mixing of *d_xy_* and *3a_2u_* occurs in the lower D_2d_ symmetry, where both orbitals exhibit D_2d_ symmetry and transform in the manner of *B_2_*.

(26) $$\left|g_{0x}\rangle =\right|\psi_{1x}\rangle {=}^{2}|{\left(3e_{x}-d_{xz}\right)}^{4},{\left(d_{xz}+3e_{x}\right)}^{3},{\left(3b_{2}-d_{xy}\right)}^{2},{\left(d_{xy}+3b_{2}\right)}^{2}\rangle \;\left|g_{0y}\rangle =\right|\psi_{1y}\rangle {=}^{2}|{\left(3e_{y}-d_{yz}\right)}^{4},{\left(d_{yz}+3e_{y}\right)}^{3},{\left(3b_{2}-d_{xy}\right)}^{2},{\left(d_{xy}+3b_{2}\right)}^{2}\rangle \;\left|g_{1}\rangle =\right|\psi_{2}\rangle {=}^{2}|{\left(3e_{y}-d_{yz}\right)}^{4},{\left(d_{\pi }+3e\right)}^{4},{\left(3b_{2}-d_{xy}\right)}^{2},{\left(d_{xy}+3b_{2}\right)}^{1}\rangle \;\left|g_{2}\rangle =\right|\psi_{3}\rangle {=}^{2}|{\left(3e-d\pi \right)}^{4},{\left(d_{\pi }+3e\right)}^{4},{\left(3b_{2}-d_{xy}\right)}^{1},{\left(d_{xy}+3b_{2}\right)}^{2}\rangle \;\left|g_{3x}\rangle =\right|\psi_{4x}\rangle {=}^{2}|{\left(3e_{x}-d_{xz}\right)}^{3},{\left(d_{xz}+e_{x}\right)}^{4},{\left(3b_{2}-d_{xy}\right)}^{2},{\left(d_{xy}+3b_{2}\right)}^{2}\rangle \;\left|g_{3y}\rangle =\right|\psi_{4y}\rangle {=}^{2}|{\left(3e_{gy}-d_{yz}\right)}^{3},{\left(d_{yz}+e_{y}\right)}^{4},{\left(3b_{2}-d_{xy}\right)}^{2},{\left(d_{xy}+3b_{2}\right)}^{2}\rangle \;\;\;\left|Q_{x}^{0}\rangle =sin\nu \cdot^{2}\right|{\left(3b_{2}-d_{xy}\right)}^{1},4e_{x}^{1},{\left(d_{\pi }+3e\right)}^{3}\rangle +cos\nu \cdot |3b_{1}^{1},4e_{y}^{1},{\left(d_{\pi }+3e\right)}^{3}\rangle \;\left|Q_{y}^{0}\rangle =cos\nu \cdot^{2}\right|{\left(3b_{2}-d_{xy}\right)}^{1},4e_{y}^{1},{\left(d_{\pi }+3e\right)}^{3}\rangle -sin\nu \cdot |3b_{1}^{1},4e_{x}^{1},{\left(d_{\pi }+3e\right)}^{3}\rangle \;\left|B_{x}^{0}\rangle =cos\nu \cdot^{2}\right|{\left(3b_{2}-d_{xy}\right)}^{1},4e_{x}^{1},{\left(d_{\pi }+3e\right)}^{3}\rangle -sin\nu \cdot |3b_{1}^{1},4e_{y}^{1},{\left(d_{\pi }+3e\right)}^{3}\rangle \;\left|B_{y}^{0}\rangle =sin\nu \cdot^{2}\right|{\left(3b_{2}-d_{xy}\right)}^{1},4e_{y}^{1},{\left(d_{\pi }+3e\right)}^{3}\rangle +cos\nu \cdot |3b_{1}^{1},4e_{x}^{1},{\left(d_{\pi }+3e\right)}^{3}\rangle$$

Note that, for the sake of brevity, only the unpaired orbitals are listed in the description of the Q^0^- and B^0^-states. The admixture of *d_xy_*- into *b_2_*-orbitals lowers the energy of the ground state, while states with (*d_xy_* + *b_2_*) orbitals move closer to (*d_π_* + *3e*). This makes the thermal occupation of the state |*g_1_>* more likely. The lowering of the ground-state energy leads to the experimentally observed redshift of the optical spectrum of the ruffled metalporphyrins [73].

While we are focused on the effect of ruffling, here, it should be noted that saddling (*B_2u_*) perturbations can mix *d_xy_*- with *3a_1u_* (*B_1_* in D_2d_)-orbitals, thus leading to the further destabilization of the former. Doming has *A_2u_* symmetry and can therefore mix $d_{z^{2}}$- and *3a_2u_*-orbitals (*B_2_* in D_2d_). This is irrelevant for low-spin ferric complexes, but it becomes relevant in all high-spin systems, irrespective of whether they are in the ferric or ferrous state.

How do all these perturbations affect the redox potential and the electron transfer properties of cytochrome c derivatives? I start with the discussion of heme ruffling. The respective deformation lowers the symmetry of the macrocycle from D_4h_ to D_2d._ For ferricytochrome c, the ground state |*g_0_*> exhibits *^2^E*-symmetry. The symmetry of the first excited state |*g_1_*> becomes *^2^A_2_*, which is lower in energy than it is in D_4h_, since ruffling destabilizes *d_xy_*, so that less energy is required to move an electron to *d_π_*. I ignore quartet states in this discussion. In the case of ferrocytochrome c, the ground state symmetry is ^1^A_1g_, which becomes ^1^A in D_2d_, since there is no hole through which the symmetry can be affected. The first excited state which involves an electron transfer from $d_{\pi }$ to $d_{z^{2}}$ transforms in the manner ^1^*E_g_* in D_4h_ and *E* in D_2d._ Electron transfer can be expected to involve an electron that occupies the higher-lying *d_π_*-orbital (most likely *d_yz_*). Hence, from a group theoretical point of view, ruffling should not affect the ionization energy of ferrocytochrome c in the zeroth order, since neither ruffling nor saddling can mix *d_π_* with the occupied porphyrin orbitals in a D_4h_ reference frame. However, the situation is different in D_2d_, where the *d_π_*-orbitals and the third-highest porphyrin orbitals transform as *E*. Since the direct product of the two orbitals can be written as *A_1_* + *A_2_* + *B_1_* + *B_2_*, practically any deformation with a one-dimensional representation can mix the two orbitals. Within the D_4h_ reference system, such a first-order effect can be ascribed to the matrix elements of vibronic coupling operators of the type $\partial {\hat{V}}^{\Gamma }/\partial q_{k}^{\Gamma^{\prime }}$. Any combination of *ungerade* electronic perturbations and vibrational modes will allow for the mixing of *E_g_* orbitals. Taken together, this group theoretical reasoning suggests that ruffling or any other out-of-plane deformation destabilizes the reduced state, because the amount of energy gained by filling the hole in the states |*g_0_>* and |*g_1_>* is decreased more than the energy required to remove an electron from the ground state of the reduced heme. Therefore, the reduction potential decreases, which is in line with experimental observations of Bren and coworkers [86,103]. DFT calculations backed by NMR results arrived at a similar conclusion in that their results suggest a destabilization of all ‘*t_2g_*’-orbitals by ruffling, though to a different extent [56]. As one would expect from the above reasoning, the effect is dominant for *d_xy_*.

As outlined above, electronic *B_1g_* perturbation causes a split of the (*d_xz_* + *3e_gx_*)- and (*d_yz_* + *3e_gy_*)-orbitals and, thus, of the *E_g_* ground state of the oxidized protein. If one chooses D_2d_ as the reference system (i.e., taking into account the influence of ruffling), the two orbitals are denoted as (*d_xz_* + *3e_x_*) and (*d_yz_* + *3e_y_*). Together, they exhibit *E* symmetry. The electronic perturbation now exhibits *B_1_* symmetry and reduces the symmetry of the heme from D_2d_ to D_2_. In this new point group, the above orbitals should be written as (*d_xz_* + *3b_2_*) and (*d_yz_* + *3b_3_*). This splitting can be substantial. In horse heart cytochrome c, for instance, it amounts to nearly 60% of the axial field splitting (ca. 440 cm^−1^). Thus, the higher-lying state (generally, the (*d_yz_* + *3b_3_*)-containing |*g_0y_*> state) lies 220 cm^−1^ above the level of the unperturbed D_4h_ symmetry. The first excited electronic state |*g_1_*> exhibits *B_2_* symmetry in D_2d_. Rhombic perturbation cannot mix |*g_0_*> and |*g_1_*>. In the reduced state, the ground state is a singlet of *A_1_* symmetry, while the first excited state exhibits *E* symmetry (*vide supra*), which splits into *B_2_* and *B_3_* in D_2_. Any occupation of this state at room temperature should be very low. As a consequence, neither the electronic *B_1_* nor *B_2_* perturbations of a heme in D_2d_ symmetry affects the ground state of the reduced heme. Altogether, these perturbations thus destabilize the oxidized state, thus increasing the redox potential.

Vibronic perturbation can affect the electronic states differently. As one can read from Equation (17), vibronic perturbation along a normal coordinate *q_k_* can practically affect all the electronic states in the presence of an electronic perturbation of the same symmetry, since the respective operator transforms in the manner of *A_1g_* in D_4h_. Even though the respective matrix elements can be on-diagonal in the pure electronic basis set, they are off-diagonal in a vibronic basis, since the operator $\partial {\hat{V}}^{\Gamma }/\partial q_{k}^{\Gamma^{\prime }}$ has to be multiplied with the operator ${\hat{q}}_{k}^{\Gamma^{\prime }}$, which reflects the mixing of the vibrational states (which can involve several states for normal modes of low wavenumbers). Hence, the energy contribution is second-order. Since the matrix elements of $\partial {\hat{V}}^{\Gamma }/\partial q_{k}^{\Gamma^{\prime }}\cdot {\hat{q}}_{k}^{\Gamma^{\prime }}$ can be in the range of, or even exceed, the vibrational wavenumbers, these perturbations cannot be treated with Rayleigh–Schrödinger perturbation theory. Obviously, these perturbations lead to the breakdown of the Born–Oppenheimer approximation. The most prominent vibronic perturbation that affects the ground state is that of *B_1_* symmetry, and this causes a JT distortion of the ground state of ferricytochrome as long its symmetry is *^2^E*. As the above electronic perturbation is of the same symmetry, it adds to the splitting of the ground state and, thus, a destabilization of the oxidized state, thus increasing the redox potential.

I complement this section by briefly mentioning that the results of the combined NMR and DFT studies suggest that ruffling deformations decrease the electronic coupling between cytochrome c and its redox partners [59]. As one expects from the canonical Marcus theory, this reduces the rate of the electron transfer. The reduction in the coupling energy results from a decrease in the unpaired electron spin density and, thus, a higher delocalization of the electron density of the iron atom. This is an interesting result in that it suggests that the mixing of *d_π_* with *3e_g_* is, to some extent, neutralized by the mixing of *d_xy_* with *3a_2u_.*

## 7. Summary and Outlook

It was the goal of this review article to provide an overview of the protein- and ligand-induced perturbations that affect the heme group of cytochrome c type proteins. For a long period of time, the electronic structures of the heme macrocycle and of the central iron atom were discussed independently. The four-orbital model of Gouterman was based on optical absorption data, while the heme iron was explored through EPR and Mössbauer spectroscopy. While the very early EPR studies of cytochrome c and other heme proteins revealed that the ligand field of the heme iron has a rhombic symmetry absorption, the resonance Raman data were generally interpreted within the framework of a heme macrocycle exhibiting D_4h_ symmetry, even though the depolarization ratio dispersions clearly showed that this notion is incorrect. As a matter of fact, the invalidity of the high-symmetry approach had already become apparent from the NMR experiments of Wüthrich and colleagues, which revealed how the orientation of the methionine ligand affects the spin distribution of the heme macrocycle. The more recent works of Walker, Bren and coworkers must be credited not only for arriving at a more comprehensive picture of the electronic structure of the cytochrome derivatives but also for demonstrating that symmetry-lowering deformations are biologically relevant.

While the work of Bren and coworkers focused on out-of-plane deformations, which were well characterized on a quantitative level by the pioneering work of Jentzen and Shelnutt, this review argued that in-plane deformations deserve some consideration as well. This article invoked group theoretical arguments to provide a comprehensive understanding of the electronic (vibronic) structure of the heme group in cytochrome c derivatives. An emphasis was placed on elucidating the relationships between electronic-perturbing potentials and the induced heme distortions.

Finally, I would like to briefly refer to the paper of Galianto et al. that has not been discussed thus far [104]. These authors made use of nuclear resonance vibrational spectroscopy to explore vibrational spectra involving the motions of the heme iron, which elude traditional spectroscopy methods, such as Raman and IR. The main result of this study was that the normal modes are actually not restricted to the heme group but involve, in particular, vibrations of the CxxCH linker and the iron ligands. These vibrations cover the region between 250 and 500 cm^−1^_._ It is very likely that the vibrational dynamics revealed by this study are of great relevance, helping to foster a thorough understanding of the respective electron transfer processes.

This article focused on cytochrome c, but various studies of other heme proteins revealed the influences of in-plane and out-of-plane deformations on, e.g., peroxidase activity and ligand binding. However, a full and comprehensive account of the ways in which heme deformations tune biological activities still has yet to be developed.

## Data Availability

Not applicable.
